# Time-series RNA-Seq transcriptome profiling reveals novel insights about cold acclimation and de-acclimation processes in an evergreen shrub of high altitude

**DOI:** 10.1038/s41598-022-19834-w

**Published:** 2022-09-16

**Authors:** Nikita Rathore, Prakash Kumar, Nandita Mehta, Mohit Kumar Swarnkar, Ravi Shankar, Amit Chawla

**Affiliations:** 1 Environmental Technology Division, CSIR-Institute of Himalayan Bioresource Technology (CSIR-IHBT), Palampur, H.P India; 2 Biotechnology Division, CSIR-IHBT, Palampur, H.P India; 3 Academy of Scientific and Innovative Research (AcSIR), Ghaziabad, 201002 India; 4 Studio of Computational Biology and Bioinformatics, The Himalayan Centre for High-Throughput Computational Biology (HiCHiCoB, A BIC of Department of Biotechnology, Govt. of India), CSIR-IHBT, Palampur, H.P India

**Keywords:** Ecophysiology, Plant ecology

## Abstract

The high-altitude alpine regions are characterized by highly variable and harsh environmental conditions. However, relatively little is known about the diverse mechanisms adopted by alpine plants to adapt to these stressful conditions. Here, we studied variation in transcriptome and physiological adjustments occurring across the year at high elevation environments in the leaf tissue of *Rhododendron anthopogon*, an evergreen shrub of Himalaya. The samples were collected at 12 different time-points, from August until snowfall in November 2017, and then from June to September 2018. It was observed that with a drop in both ambient air temperature and photoperiod towards onset of winter, the freezing resistance of plants increased, resulting in ‘cold acclimation’. Further, ‘de-acclimation’ was associated with a decrease in freezing resistance and increase in photosynthetic efficiency of leaves during spring. A considerable amount of variation was observed in the transcriptome in a time-dependent sequential manner, with a total of 9,881 differentially expressed genes. Based on gene expression profiles, the time-points could be segregated into four clusters directly correlating with the distinct phases of acclimation: non-acclimation (22-August-2017, 14-August-2018, 31-August-2018), early cold acclimation (12-September-2017, 29-September-2017), late cold acclimation (11-October-2017, 23-October-2017, 04-November-2017, 18-September-2018) and de-acclimation (15-June-2018, 28-June-2018, 14-July-2018). Cold acclimation was a gradual process, as indicated by presence of an intermediate stage (early acclimation). However, the plants can by-pass this stage when sudden decrease in temperature is encountered. The maximum variation in expression levels of genes occurred during the transition to de-acclimation, hence was ‘transcriptionally’ the most active phase. The similar or higher expression levels of genes during de-acclimation in comparison to non-acclimation suggested that molecular functionality is re-initiated after passing through the harsh winter conditions.

## Introduction

Various mild to severe environmental stress factors in high elevation environments [such as low ‘mean annual temperature’, low ‘partial pressure of gases’, high ‘diurnal thermal fluctuations’, high ‘wind velocity’, high intensities of ‘solar radiation (including high UV-B radiation)’] influence plant growth and development. The short growing season at higher elevations is another limiting factor that affects the growth rate in plants and constrains reproductive success^1^. Further, the temperature and photoperiod gradient i.e., ‘cold air temperatures and shorter photoperiod’ during winter and ‘warmer air temperatures and long photoperiod’ in summer, plays a key role in defining the plant performance^2,3^. The transition from a warmer and more stable environment (during spring and summer) to colder and harsher conditions (towards winters) could be detrimental to the plant’s survival. At high altitudes, the temperature may fall below the freezing point in the autumn and winter season, which may lead to intra-cellular ice formation and, ultimately, cell death in plants^4^. The plants facing this transition from optimal to stressful conditions have developed adaptation (‘seasonal acclimation’) to successfully persist under such a variable environment^2^.

Plants, being sessile, are constantly exposed to environmental variations throughout their life-span^5^. To persist in challenging environments, plants develop diverse adaptive strategies including morphological as well as physiological adjustments, which are ultimately the result of alterations in underlying molecular processes^6,7^. Whole genome transcriptome approach can help to understand the underlying molecular processes and regulatory programs^8–10^. For instance, a study provided evidence of key role of annual temperature changes in defining seasonal oscillations of gene expressions and adaptation to seasonal environments in a perennial plant *Arabidopsis halleri* using this approach^2^. Another study revealed that the interaction between day length and temperature controls the annual transcriptome dynamics in Japanese cedar (*Cryptomeria japonica*) under natural conditions^11^. Similarly, an increase in expression level of diverse genes encoding proteins with functions such as biosynthesis of sugars and lipids, transcriptional activators related to C-repeat/dehydration-responsive element binding factors etc., have been identified during the cold acclimation process^12,13^. However, most of these molecular features, identified to be involved in plant’s response to varying environment, have been deciphered through experimental studies conducted in the laboratory^14–16^. It is not clear when and which of these molecular systems are active in plants occurring in the natural environment. A few existing studies have analysed the expression of certain key genes in a natural environment^2,17^, however, the present understanding of how the molecular machinery of plants functions under seasonally varying environmental conditions (i.e., favourable to harsh) remains limited.

Recently, the transcriptome dynamics in plants is being studied utilizing the samples collected from natural environments in a time-series manner to understand the molecular regulation that takes place during the seasonal environmental responses^2^. For example, a study uncovered distinct gene family expansions (e.g. terpene synthases) in *Cupressus gigantea*, a long-lived species, and demonstrated their importance in context to adaptability to seasonal ‘environmental cues’^18^. In such cases, the time-series data allows uncovering the relative dynamics of transcriptional changes. Specifically, it allows for determining change in the expression of genes (up/down-regulation) during different time-points, along with the magnitude and duration of that change. Although “time-series” approach has many advantages, the existing studies on transcriptome changes in plants in response to environmental variability have been based on fewer or widely spaced time-points (e.g. up to 3 or 4 sampling time points in a year). Therefore, a complete understanding of transcriptome changes in plants occurring in varying environment is lacking.

Furthermore, the understanding of plant adaptive strategies against different types of stresses comes through studies conducted on only a few species [such as *Arabidopsis thaliana* or crop plants (e.g. rice, maize, wheat, chickpea etc.)]^7,19–22^. Based on extensive studies on these species, many important advances have been made in plant biology (e.g. processes involved in plant growth regulation, photosynthesis etc.)^20^. But, since these are annual plants which encounter only optimal growth conditions, this limits our ability to get insights into adaptive responses of various species belonging to other growth forms (e.g. woody perennials) and inhabiting totally different and harsher climates (e.g. at high altitudes). The woody species occurring at higher elevations are either deciduous or evergreen. The deciduous species also complete their growth cycle during favourable environmental conditions and therefore, are exposed to less environmental variability in comparison to the evergreen species. The latter, on the other hand, faces the full extent of this variability throughout the year and are expected to have evolved specialized strategies for persistence under highly variable environments. Further, the evergreen perennials can be classified into two major types: needle-leaved (conifers) or broad-leaved. The adaptive responses of a few needle-leaved species (*Pinus* spp., *Picea* spp. etc.) to environmental variability in natural environments have been studied to a considerable extent^23–27^. A few studies have also been undertaken on broad-leaved evergreen species occurring in temperate environment, such as *Rhododendron* spp.^13,28–31^, *Buxus sempervirens*^32^ etc*.*, but the focus has been less, as compared to the needle leaved species*.* Given the ‘large differences in anatomy’ and ‘evolutionary distinctness’ between needle-leaved and broad-leaved woody perennials (separated ~ 300 million years ago)^11,33^, the broad-leaved species are, however, expected to show relatively different response patterns to environmental variability. Further, the broad-leaved evergreen species, such as the dwarf shrubs, which occur at high elevations, are exposed to much harsher conditions than the temperate conifers. Therefore, the broad-leaved woody perennials found in alpine regions are functionally very distinct from other species (i.e., herbaceous annuals or woody gymnosperms). A few existing studies utilizing broad-leaved woody perennials have covered various aspects of ecological processes such as leaf senescence, floral bud development etc*.*^34,35^; however, there are many questions which remain un-addressed.

Here, we explored the molecular regulation that takes place on a seasonal basis in *Rhododendron anthopogon* D. Don, an evergreen broad-leaved shrub of high-altitude alpine environments, using a time-series transcriptome approach. *R. anthopogon* occurs in open sunny habitats and forms small to large patches in the alpine meadows. It grows at high elevations (3000–5000 m a.s.l) of Himalaya. It is one of the dominant species of alpine regions of Himalaya^36^ and has a broad niche width^37^. It is also among those shrub species which are reported to occur at the highest elevations in Himalaya and form the shrub-lines. In its natural environment, this shrub often gets subjected to substantial environmental variations along spatio-temporal gradients. All these characteristics indicate well adaptability of this species to grow in the high-altitude environment and make it enticing for molecular and functional analysis, which could provide valuable insights into various aspects of plant acclimation biology.

Thus, studying adaptive responses of species growing in their natural habitat could provide invaluable information about how plants prepare themselves to persist under changing environmental conditions. The common approach to investigate plant responses to environmental variability has been to resample the same plant population (s) and directly make comparison between them^38^. Due to its evergreen nature, the foliage of *R. anthopogon* remains functional for more than one annual cycle, thus enabling repeated measurements on a given leaf type to be conducted across multiple time-points. So, this alpine dwarf shrub could be utilized to understand plant adaptation strategies in harsh environmental conditions of high elevations.

We expected that, as the plants are subjected to a range of environmental conditions during their entire growing period, there would also be varying responses at molecular level (i.e., it would involve different gene expression patterns). Specifically, the biological processes involved in primary metabolism (e.g. photosynthesis) would slow down with the onset of low temperature conditions, whereas, those associated with synthesis of biomolecules, which play an important role in conferring cold tolerance (e.g. dehydrins), would be increased. Based on these hypotheses, we aimed to elucidate how transcriptome is reshuffled on a seasonal basis in response to changing environmental conditions in natural alpine environments.

## Material and methods

### Study site and plant sampling

The leaves of *R. anthopogon* were collected from the site established at an elevation of 3990 m amsl, at Rohtang, which lies in the western Himalayan region in India. Plants used in this experiment were identified by a plant identification expert. The voucher specimen was deposited in the CSIR-IHBT, herbarium [Acronym PLP; Palampur (H.P.) India 176061], vide voucher number: PLP18623. The collection of plant materials complied with institutional, national, or international guidelines and legislation.

To unravel how plant physiology and transcriptome reshuffle on a seasonal basis in response to changing environmental factors, leaves developed in the same year prove to be the best biological material. Since *R. anthopogon* is an evergreen species, performing repetitive measurements and experimentations for at least one year was possible with newly developed leaves only. As observed in the field, new leaves of *R. anthopogon* start developing soon after the snowmelt (May–June), become fully expanded during August, pass through an overwintering phase, and thereafter, the same leaves undergo a de-acclimation process. Because of these important observations, we chose to utilize newly developed leaves from plants growing in the natural alpine habitat experiencing seasonal and environmental variations in real-time. To avoid any effects related to genotype, plants from a population growing in the same plot were marked and sampled during all the sampling time-points. Accordingly, the leaf tissue sampling was initiated in the third week of August 2017 and continued till November in the same year. Afterward, the plants got covered by a thick layer of snow (~ 240 days), and hence, further sampling was not possible due to the inaccessibility of the study site until snowmelt. The sampling was resumed in mid-June (after the snow melt) and continued till September 2018. Samples were collected at six time-points between August and November 2017, and six samples were collected between June and September 2018. Sampling was undertaken at an interval of 2–3 weeks for a total of 12 different time-points: 22-August-2017, 12-September-2017, 29-September-2017, 11-October-2017, 23-October-2017, 04-November-2017, 15-June-2018, 28-June-2018, 14-July-2018, 14-August-2018, 31-August-2018 and 18-September-2018. The time-points and interval period (~ 2–3 weeks) between them depended on the probability of getting a cloud-free sunny day for sampling purposes at the study site. Also, the challenging terrain in the natural habitat was an important limiting factor in the feasibility of plant sampling at more frequent intervals.

Three independent biological replicates were collected at each time-point (one replicate from one plot). A similar sampling procedure for collection of replicates, as used in the previous study was followed^39^. Repetitive sampling and plant trait measurements were done from the same plots and from the same population. The sampling was always undertaken between 9.30 a.m. and 11.30 a.m. on clear sunny days. The collected leaves were snap frozen in liquid nitrogen, transported to the laboratory, and stored at − 80 °C until processed further for transcriptome analysis. In principle, RNA-Seq is an important tool and can yield valid information on adaptation of plants to harsh environmental conditions. Important insights can be gained by taking measurements in a time-series manner, as has been undertaken in the present study, and complementing with physiological datasets. Such a sampling strategy, utilizing RNA-Seq transcriptome analysis, could thus yield a reasonable dataset to deduce the changes with certainty.

Temperatures for air and soil were recorded at the study site for the entire study period using temperature data loggers (M-Log5W, GEO Precision). The monthly average air temperature was found to be ranging between − 9.8 (± 3.7) and 9.4 °C (± 2.4), with January being the coldest month (minimum of − 18.2 °C), and July as the hottest (maximum of 18.4 °C). From October 15 till the end of April in the following year, the air temperature consistently remained near 0 °C or lesser^39^. The monthly average temperature of the soil varied from − 5.4 (± 1.2) to 10.9 °C (± 1.9), with January as the coldest (minimum of − 8.9 °C) and August as the warmest month (maximum temperature of 15.8 °C). As with the air temperatures, the soil temperatures also dropped below 5 °C on October 15, 2017, and remained less than 0 °C after November 19, 2017, till May 15, 2018. The total annual precipitation (mostly snow) was approximately 1024 mm, with November as the driest month (26 mm) and March being the wettest month, with 148 mm of precipitation (mostly snow). From June to September, precipitation occurred in the form of rainfall, with July as the wettest month (107 mm). The photoperiod (h day^−1^) was estimated by extrapolating from the latitudinal value of the study site, using the “insol” package in R. It was found to be ranging between 10.6 h day^−1^ (November 4, 2017) and 14.1 h day^−1^ (15-June-2018) during the study period^39^.

### Leaf physiological measurements

Freezing resistance of the leaves was measured by electrolyte leakage method (see^39^). Briefly, leaves removed from twigs were washed in distilled water to remove the debris and surface electrolytes. The leaves were dab-dried properly. 10–12 leaves weighing approximately 300 mg were inserted into plastic tubes. The leaves were given temperature treatment, beginning with an initial temperature of 4 °C and reducing in a gradual manner at a rate of 3 °C h^−1^ until reaching the ‘freezing temperature treatment’ (0 °C, − 5 °C, − 10 °C, − 15 °C, − 20 °C, − 25 °C, − 30 °C, − 35 °C, − 40 °C, − 50 °C, − 60 °C and − 70 °C) in a test chamber. The “freezing treatment” was maintained for 1 h. Relative electrical leakage (REL) % was estimated for each temperature treatment. ‘LT_50_’ was used to estimate the level of freezing resistance during each sampling time-point. LT_50_ was estimated as the temperature of the inflection point, calculated by applying a sigmoidal differential equation for curve fitting on the REL % values calculated for each temperature treatment, using the “nplr” package of R. The freezing resistance measurements for a sampling point were done for a total of five replications. All the analyses were performed using R 3.6.1 statistical software^40^.

Further, various leaf gas exchange parameters were measured in the field using a portable photosynthesis system (Li-Cor Inc., Li-6400XT, Lincoln, Nebraska, USA). The light response curves were prepared to determine the saturating irradiance level [~ 2000 µmol (photon) m^−2^ s^−1^] for *R. anthopogon* at the study site. Within the leaf chamber, the temperature was set to be the same as the ambient temperature during measurement. Similarly, relative humidity (RH) in the leaf chamber was equilibrated with the current outside air RH, and the measurements were recorded at ambient CO_2_ concentration (µ mol m^−2^ s^−1^). The fully expanded leaves were clamped into the cuvette of gas analyzer. Once a steady-state gas exchange rate was reached (usually 20 min after clamping the leaf), net CO_2_ assimilation rate (P_N_) [µmol(CO_2_)m^−2^ s^−1^], transpiration rate (E) [mmol(H_2_O)m^−2^ s^−1^] and stomatal conductance (G_S_) [mol(H_2_O)m^−2^ s^−1^] were estimated. Gas exchange measurements were used to estimate photosynthetic water use efficiency (WUE): WUE = P_N_ [µmol(CO_2_)m^−2^ s^−1^]/E [mmol(H_2_O)m^−2^ s^−1^]^41^. These measurements were taken during three sampling time-points (i.e., 15-June-2018, 31-August-2018 and 18-September-2018). All the leaf gas exchange measurements were done for a total of five replications. These measurements were taken between 9:00 and 11:00 a.m.

The mean values of different gas exchange parameters of *R. anthopogon* were compared among the three time-points using one-way ANOVA. A Tukey’s *post-hoc* test was then performed to examine significant differences in means for pairwise comparisons between each possible combination of the time-points (*p* ≤ 0.05). All the analyses were performed using R 3.6.1 statistical software^40^.

### Transcriptome profiling

#### RNA extraction, RNA-Seq library preparation and sequencing, and De novo assembly

Total RNA was extracted^42^ and, the quality and quantity of total RNA was analysed using NanoDrop spectrophotometer (PerkinElmer, DropSense 96, Unchanged Labs, USA) and Qubit 2.0 Fluorometer (Life technologies, USA). The quality of extracted RNA was also assessed by resolving them on a formaldehyde agarose gel (1.2%). A total of thirty-six (12 time-points × 3 replicates) RNA-Seq libraries were prepared using QIA-Seq Stranded mRNA library kit (Qiagen, Netherlands) following the manufacturer’s instructions. The libraries were quantified using Qubit™ dsDNA BR assay kit in a Qubit 2.0 fluorometer (Life technologies, USA). The quality of libraries was checked using a DNA 1000 chip on Agilent 2100 Bioanalyzer system (Agilent technologies, USA). About 400 pico-molar of pooled-denatured library along with Phix control (1% spike-in) was loaded onto the S2 flow cell for clonal amplification followed by paired-end (PE) sequencing [150 × 2 base pairs (bp)] on Illumina Novaseq 6000 sequencing system (Illumina Incorporation, USA) as per manufacturer’s instructions. The high-quality reads were stored in FASTQ format, de-multiplexed and subsequently used for de novo assembly (Supplementary Fig. S1). The details of ‘de novo assembly’ and ‘functional annotation and classification’ are provided in Supplementary information.

#### Differential expression analysis

To quantify the expression of unigenes, high quality reads of each of the samples were separately mapped onto the assembled unigenes using Bowtie2 version v2.2.5. Counts of uniquely mapped reads were calculated using ‘bash shell script’. The count files were applied to edgeR as ‘matrix of mapped reads count’ for each time-point for scaling raw count values. FPKM values were calculated (based on read count) for determining the expression level of unigenes using RSEM-1.3.1 software package^43^.

The FPKM values (after normalization) of expressed unigenes were clustered using agglomerative hierarchical clustering by complete-linkage method with Pearson correlation using R function ‘hclust’. Trimmed Mean of M-values (TMM) was used as a scaling normalization method while performing clustering. For piloting heatmap, ‘heatmap.2’ function of R was used. The clustering of genes was performed using different R packages (“edgeR”, “limma”, “RcolorBrewer”, “mixOmics”, “HTSFilter”, “gplots” and “dendextend”). In the cluster dendrogram, a replicate of a time-point (i.e., 31-August-2018) appeared as an outlier, and therefore this replicate was removed from the subsequent analysis. Following this, pair-wise comparisons of normalized counts were performed in all possible combinations among different sampling time-points to identify differentially expressed genes (DEGs), using “edgeR v3.14.0” (a Bioconductor package for differential expression analysis of gene expression data)^44,45^. A generalized linear model was used to test for statistical significance in the expression of a gene among different time points [*p* value ≤ 0.01 and absolute log_2_ (fold change) ≥ 2]. Further, to determine relationship between transcriptome profiles of *R. anthopogon* for the 12 time-points, hierarchical clustering was performed using DEGs dataset. Thus, the time-points could be grouped into four distinct clusters. To identify DEGs between these respective clusters, pair-wise comparisons of normalized counts among the four clusters were performed [*p* value ≤ 0.01 and absolute log_2_ (fold change) ≥ 2].

Further, to identify significantly enriched GO terms between different cluster combinations, DEGs were subjected to singular enrichment analysis using ‘AgriGO’, a web-based tool for GO analysis (http://bioinfo.cau.edu.cn/agriGO/). GO enrichment analysis was performed using Hyper-geometric test with Bonoferroni analysis method (*p* ≤ 0.05). Similarly, KEGG Pathway analysis was performed to identify significant pathways between the different cluster combinations. A Fisher’s exact test was used to find significantly enriched pathways. A threshold of significance was set at *p* value ≤ 0.05 for KEGG enrichment.

#### Validation of DEGs using quantitative real time PCR (qPCR)

For the validation of genes, which were identified through RNA-seq, qPCR was undertaken using RT-PCR system (Thermofisher Scientific, Applied Biosystems QuantStudio 3-flex RT-PCR, USA). DEG-specific primers were designed using the Primer Express 3.0 software (list of primers is given in Supplementary Table S1). As four distinct phases (with similar gene expression in time points in a phase) were identified in the transcriptome analysis, therefore, validation was also done using one representative time-point from each phase.

Total RNA (5 µg) was used for the synthesis of cDNA, using a reverse transcription kit (Thermofisher Scientific, Verso cDNA synthesis kit, India). The qPCR of cDNA samples was performed in triplicates. In a total volume of 10 µl reaction, 5 μL of 2× SYBR Green PCR mix (Thermofisher Scientific, PowerUp™ SYBR™ Green Master Mix, India) was combined with 2.5 μL of cDNA template (synthesized from 5 μg of RNA followed by 100× dilution), 0.5 μL of each primer (10 mM) and 1.5 μL of RNase—free water. In addition, a non-template control was included for each replicate with RNase—free water instead of cDNA templates. The qPCR program comprised of a pre-incubation step of 10 min at 95 °C, 35 cycles of DNA amplification for 15 s at 95 °C and 15 s at annealing temperature followed by extension at 72 °C for 30 s. This was followed by a ‘melting-curve program’ comprising of 95 °C for 15 s, 60 °C for 1 min and 95 °C for 15 s. Glyceraldehyde 3-phosphate dehydrogenase (GAPDH) was used as internal control. The data (Ct values) obtained through qPCR were analysed to estimate the relative gene expression between different clusters following the ΔΔCt method^46^. First, a ΔCt value was calculated as: ΔCt (target gene) − ΔCt (reference gene). Second, ΔΔCt was calculated as: ΔCt (test sample) − ΔCt (calibrator sample). These Ct values were subjected to one-way ANOVA, followed by Tukey’s *post-hoc* test for knowing the significant pairwise differences between the means (*p* ≤ 0.05).

## Results and discussion

### Plants increase their freezing resistance upon exposure to low temperature

The freezing resistance (LT_50_ values) was found to vary ranging from − 6.9 °C (14-August-2017) to − 31.7 °C (04-November-2018) over the course of study period. The freezing resistance of leaves recorded during the 12 sampling time-points has been provided in Table 1 (also see^39^). The overlap of confidence intervals around the mean was examined for comparison of LT_50_ values for the different sampling time-points. Significant differences in freezing resistance were observed across the sampling time-points (Table 1). Leaves of *R. anthopogon* collected during summer [July and August (Air temperature and photoperiod was about 9.6 °C and 13 h day^−1^ respectively)] showed marginal resistance to freezing (LT_50_: − 7 °C) and thus, are more susceptible to freezing damage. Further, as the ambient air temperature and photoperiod decreased towards the end of growing season (i.e., October and November 2017 with air temperature and photoperiod of about − 1.1 °C and 10.5 h day^−1^ respectively), the plants acquired the highest freezing resistance (LT_50_: − 30 °C). Interestingly, a sharp increase in freezing resistance (− 29.4 °C) was observed in September 2018, when the daily mean air temperature decreased below 0 °C due to sudden snowfall (Supplementary Fig. S2). Comparison of LT_50_ values of all the leaf samples of *R. anthopogon* showed that cold de-acclimation occurred after the snowmelt during early spring in June (LT_50_: − 13.4 °C) with an increase in air temperature and photoperiod. These results demonstrated that *R. anthopogon* plants exhibit lowered freezing resistance during the warmer months [hence, these time-periods were referred as non-acclimation (NA)], progressively develop greater freezing resistance during the onset of winter season (hence, referred as cold acclimation) followed by an intermediate level of freezing resistance during the spring [hence, these time-periods were referred as de-acclimation (DA)].

**Table 1:** The estimates of LT_50_, calculated by fitting sigmoidal curve to electrolyte leakage values of temperature treatments, recorded for leaves collected during the different sampling time-points (from August 22, 2017 to September 18, 2018).

Sampling time-points	LT_50_ (°C)
22-August-2017	− 6.9
12-September-2017	− 10.3
29- September -2017	− 18.5
11-October-2017	− 19.1
23-October-2017	− 30.5
04- November-2017	− 31.7
15-June-2018	− 13.4
28-June-2018	− 7.4
14-July-2018	− 7.3
14-August-2018	− 7.2
31-August-2018	− 9.3
18-September-2018	− 29.4

**Figure 1:**
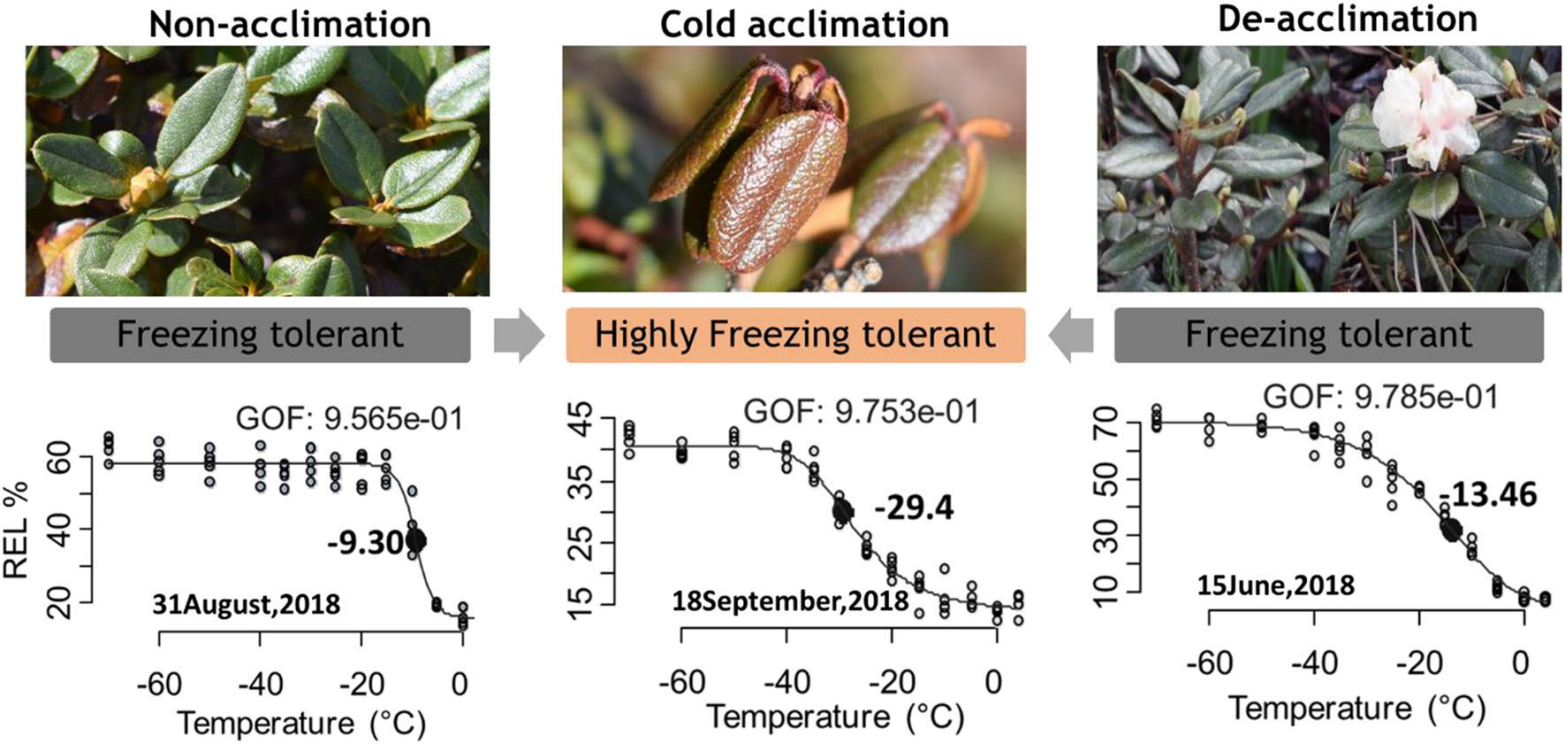
LT50 [black point (with solid fill) on the curve] calculated by fitting sigmoidal curve to relative electrolyte leakage (REL %) values recorded during the three different acclimation phases. GOF indicates ‘goodness of fit’ test values for the fitted sigmoidal curves.

During the acclimation period (i.e., late in the growing season), plants acquired the highest resistance to freezing (Fig. 1). The low electrolyte leakage (= high freezing resistance) observed during this period might be due to changes in cell wall properties (such as increase in lignification and suberization of cell walls), which provide resistance to diffusion of electrolytes from cells of the leaves to the extracellular water^47^. Moreover, high freezing resistance may also be attributed to high leaf toughness and sclerophyllous habit of this evergreen species^48^. Further, it was found that freezing resistance was the lowest during mid-summer period. This pattern could be explained by a trade-of between plant growth rates and freezing resistance, where warmer temperatures favour plant allocation to growth^49^. These observations corroborated well with earlier reports that showed a rapid increase in ‘freezing resistance’ during the transition from summer to early winter and vice versa^50^.

### Photosynthetic rates are higher during non-acclimation and de-acclimation period

**Figure 2:**
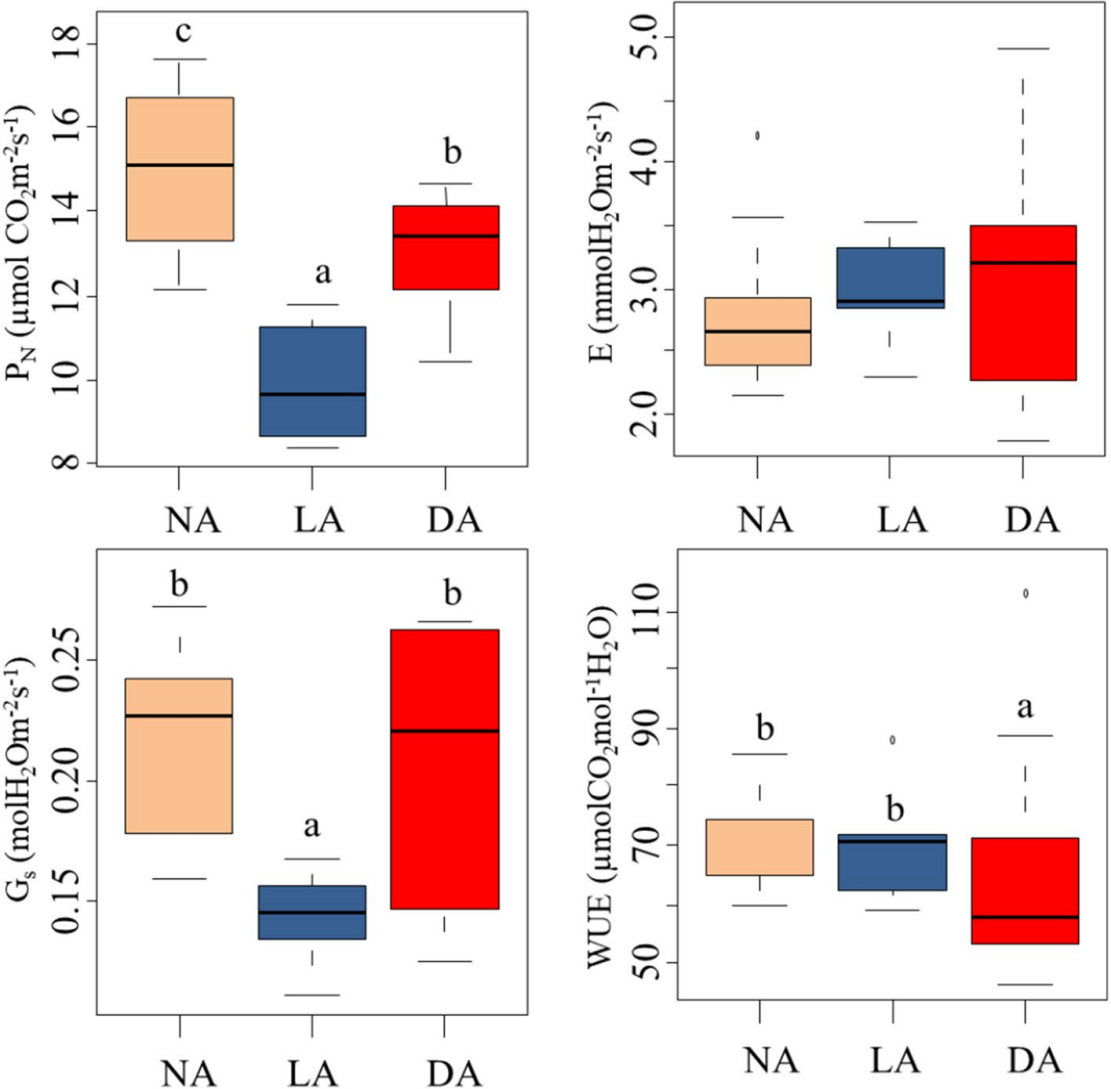
Variability in leaf gas exchange parameters of *R. anthopogon* during the three acclimation phases [i.e., Non-acclimation (31 August, 2018), Cold acclimation (18 September, 2018) and De-acclimation (15 June, 2018)]. Different alphabets (a, b, c) represent statistically significant values (*p* < 0.05) as determined by Tukey’s *post-hoc* test. P_N_ = Photosynthetic rate, E = Transpiration rate, G_S_ = Stomatal conductance and WUE = Water use efficiency.

It was found that P_N_ of *R. anthopogon* varied in the range from 8.336 to 17.64 μmol(CO_2_)m^−2^ s^−1^ and E from 2.281 to 4.912 mol(H_2_O)m^−2^ s^−1^, throughout its growing season. The G_s_ of leaves was estimated to be in the range from 0.110 to 0.265 mol (H_2_O) m^−2^ s^−1^. WUE, a ratio of P_N_ and E, varied between 52.21 and 87.68 (Table 2). The gas exchange parameters of *R. anthopogon* varied significantly among the sampling time-points [referred to here as different acclimation phases of the growing period of evergreen shrub (Fig. 2, Table 3)]. In particular, P_N_ was significantly lower on 18-September-2018 (referred as cold acclimation phase), whereas it was higher on 31-August-2018 and 15-June-2018 (referred as NA and DA phases, respectively). Similarly, Gs of leaves was significantly lower during cold acclimation in comparison to the rest of the acclimation phases (i.e., NA and DA). Further, WUE was significantly higher during cold acclimation, while it was lower during both NA and DA (*p* ≤ 0.05) (Fig. 2).

**Table 2:** Variability in leaf gas exchange parameters of *R. anthopogon* during the different acclimation phases (NA = Non-acclimation, LA = Late cold acclimation and DA = De-acclimation).

Acclimation phase	Photosynthetic rate, P_N_ [μmol(CO_2_)m^−2^ s^−1^]	Transpiration rate, E [mol(H_2_O)m^−2^ s^−1^]	Stomatal conductance, G_S_ [mol(H_2_O)m^−2^ s^−1^]	Water use efficiency, WUE
NA (31-Aug-2018)	15.89 ± 1.809	3.251 ± 0.575	0.236 ± 0.032	67.74 ± 3.222
LA (18-Sep-2018)	9.918 ± 1.529	2.980 ± 0.417	0.142 ± 0.019	70.32 ± 9.933
DA (15-Jun-2018)	13.21 ± 1.127	3.566 ± 0.872	0.217 ± 0.051	54.23 ± 2.251

Values are reported as mean ± standard deviation.

**Table 3:** Summary of one-way ANOVA results showing the effect of ‘seasonal gradient’ factor on leaf gas exchange parameters of *R. anthopogon*.

Parameter	Df	TSSq	MSSq	F value	*p* Value
Photosynthetic rate, P_N_ [µmol(CO_2_)m^−2^ s^−1^]	2	95.26	47.63	17.92	** < 0.001**
Transpiration rate, E [mmol(H_2_O)m^−2^ s^−1^]	2	0.553	0.276	0.517	0.603
Stomatal conductance, G_S_ [mol(H_2_O)m^−2^ s^−1^]	2	0.021	0.011	5.879	**0.008**
Water use efficiency, WUE [photosynthetic rate/transpiration rate]	2	956.8	478.4	6.698	**0.005**

*Df* degree of freedom, *TSSq* total sum of squares and *MSSq* mean sum of squares.

Significant values are in bold.

### Transcriptome varies in a time dependent manner and depends upon environmental conditions

**Figure 3:**
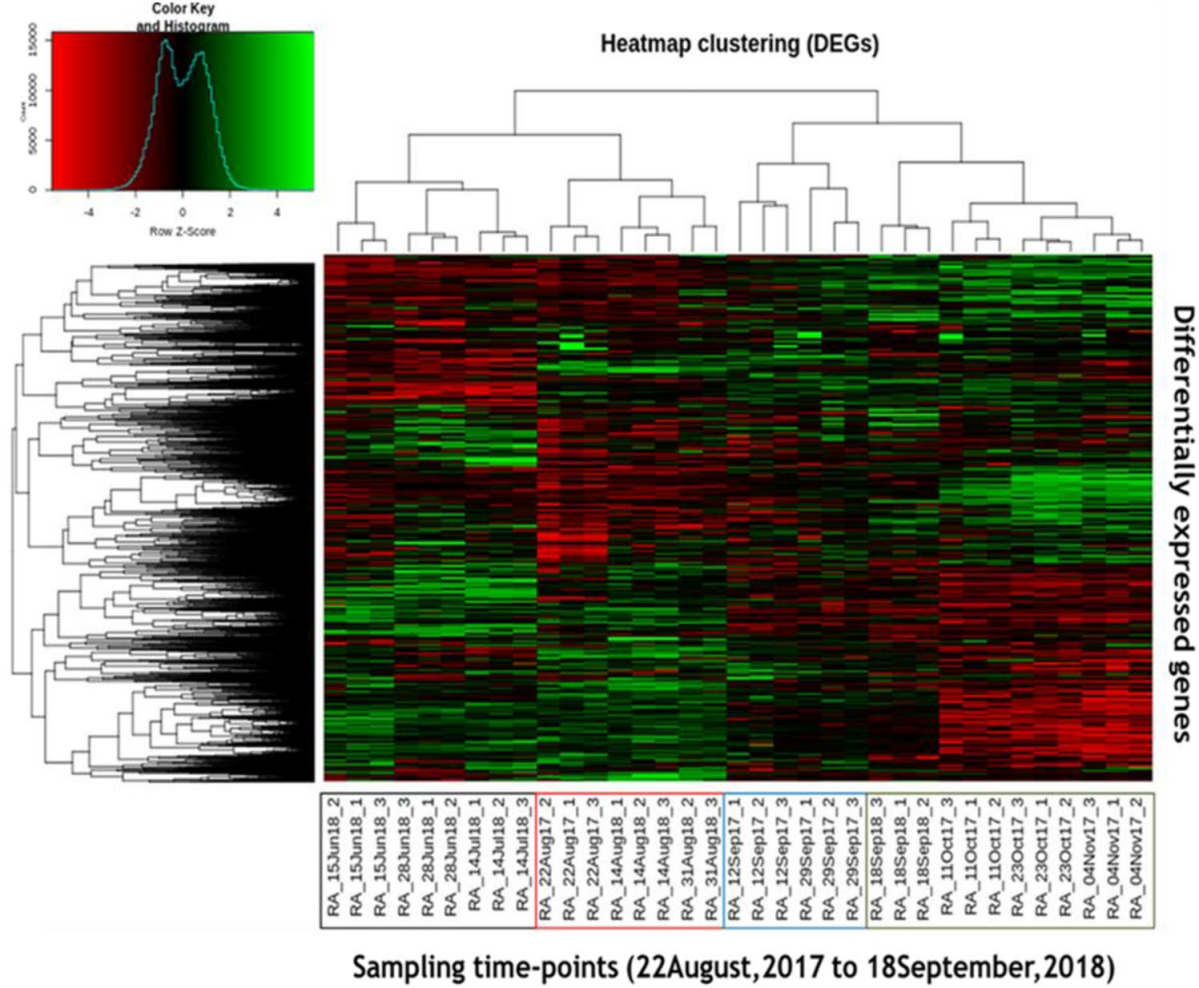
Hierarchical clustering based on expression levels of differentially expressed genes of *R. anthopogon* during the study period (from 22 August, 2017 to 18 September, 2018).

A total of 9881 genes, out of 29,096, exhibited significant differential expression in pairwise comparisons in all possible combinations of the samples (*p* ≤ 0.01 and cut-off of twofold change on log_2_ scale). The family classification of these significant differential expression genes has been provided in Supplementary Figs. S6 and S7. The hierarchical clustering of these DEGs revealed a clear grouping of time-points into four distinct clusters based on changes in ‘relative gene expression levels’ over time (Fig. 3). Here, a ‘cluster’ referred to a ‘set of time-points’ during which *R. anthopogon* have possibly similar gene expression patterns. The clustering of DEGs resulted in two main parent clades, which were further divided into two sub-clades each (Fig. 3). It was found that the samples collected during August, irrespective of the year (i.e., 22-August-2017, 14-August-2018 and 31-August-2018) clustered together, whereas the samples collected on 12-September-2017 and 29-September-2017 were found to be grouped together forming another cluster. Similarly, the samples collected during October, 2017, November, 2017 and September, 2018 (i.e., 11-October-2017, 23-October-2017, 04-November-2017 and 18-September-2018) were found to be grouped together, and those of June and July time-points (i.e., 15-June-2018, 28-June-2018 and 14-July-2018) clustered together forming a distinct cluster (Fig. 3).

Based on the period, when growth of this woody shrub is known to occur and from temperature and photoperiod records, these clusters were categorized into four phases: ‘non-acclimation’ (NA), ‘cold acclimation’ [which could be further divided into ‘early acclimation’ (EA) and ‘late acclimation’ (LA)], and ‘de-acclimation’ (DA). The ‘NA phase’ was represented by samples collected during August (2017 and 2018) and the ‘EA phase’ was represented by samples collected in September 2017. The time period during October 2017, November 2017 and in September 2018 could be referred to as ‘LA phase’. And the ‘DA phase’ was represented by samples collected during June and July (Fig. 3). The well separation of phases (viz., NA, EA, LA and DA) in the clustered heatmap suggested that the transcriptome of plant was substantially different across the four phases. This phase separation was observed to occur in a time dependent manner, as samples collected at different time-points segregated in chronological order in the clustered heatmap. It is noteworthy that variation in the transcriptome of *R. anthopogon* could be described according to the ‘changes in season’ i.e., summer (represented by NA phase), autumn (EA phase), winter (LA phase) and spring (DA phase), and hence could be termed as “seasonal acclimation” at molecular level^2^. This study was initiated when the leaf of this species got fully expanded in mid-August. Therefore, any effect will be only due to environmental conditions that vary from season to season rather than developmental changes. This was also supported by our previous study, where we showed that anatomy of leaves remains unchanged^51^. Also, the LT_50_ data clearly showed that the increased freezing resistance during late cold acclimation was not due to status change of leaves (i.e., development), but it was in response to variation in environmental factors. The freezing resistance increased maximally when the leaves of *R. anthopogon* encountered with sudden decrease in temperature in September 2018 (next year) (Table 1).

Generally, three main stages (i.e., non-acclimation, cold acclimation and de-acclimation, as represented by NA, LA and DA respectively in this study) have been reported and characterized in the previous studies^8,10,52–54^. In this study, a transitory stage (i.e., EA) was also identified during the transition from NA to LA. This transitory stage was also reported in needles of *Picea obvata*, but this was based on metabolite profiling^23^. Further, this transitory stage (i.e., EA) was not observed in 2018, as the samples collected during September 2018 (18-September-2018) did not group with the samples of September 2017, which constituted the EA stage. Rather, the September 2018 samples were grouped as a single cluster along with the samples collected during October and November in the heatmap (Fig. 3). Hence, the EA stage was either by-passed or rather a very quick transition to LA stage from NA through EA might have occurred in 2018. This observation in the transcriptome dataset was supported by the fact that the freezing resistance of leaves during September 2018 was similar to that observed in October and November, whereas it was significantly different from the values observed in September 2017 which constituted the EA stage (Table 1). Moreover, the temperature patterns recorded during September 2018 were similar (i.e., < 0 °C) to those observed in October and November 2017 (Supplementary Fig. S2). Therefore, a sharp decrease in temperature during September 2018 could have resulted in rapid cold acclimation during 2018, thereby resulting in rapid transition through EA stage. We might have missed monitoring of the quick transition to LA stage from NA through EA, due to sudden and unexpected snowfall at the study site. These results provided supportive evidence to the fact that temperature plays an important role in regulating the changes at transcript level. This is in line with the earlier reports that demonstrated the key role of temperature in controlling the annual dynamics of transcriptome in *Arabidopsis halleri*^2^. Therefore, it can be inferred that changes in transcriptome associated with cold acclimation are dependent upon the rate of change in temperature i.e., a rapid decrease in temperature leads to rapid acclimation.

Further, in the ‘clustered heat map’, it was evident that DA and NA phases were placed together in one parent clade (Fig. 3). These results suggested that transcriptome profile of *R. anthopogon* during the DA and NA phases was similar to each other, albeit with a few differences in the transcriptome. The similarity between the transcriptome during ‘DA and NA’ phases can be explained by the fact that temperature patterns during these two phases were quite similar. The air mean temperature during DA [air mean temperature observed for the clustered sampling time-points in DA cluster (sampling day and previous two days)] was 7.9 °C and, during NA, this was just slightly higher (i.e., 9.4 °C). In the transcriptome analysis, it was observed that expression levels of most of the genes were quite similar among the NA and DA phases. Considering the fact that temperature plays a prominent role in governing gene expression in plants^2^, this was an expected outcome as the temperature profile during both of these phases were similar.

On the other hand, despite some differences in gene expression patterns between EA and LA (2723 genes expressed differentially between the two phases), these two phases were found to be grouped together in the cluster dendrogram (Fig. 3). This indicated that transcription profiles during these phases were quite similar. This similarity can be explained by the sequential nature of different phases (i.e., from NA to EA to LA) and unidirectional change in temperature (decrease) during these phases. In the previous studies, it has been reported that cold acclimation is induced by a gradual decrease in temperatures in the early autumn (represented by EA phase)^8,23^. In this study, this was also supported by the fact that most of the genes which were found to be down-regulated during EA, continued to be down-regulated during the LA phase as well.

### De-acclimation is the transcriptionally most active phase, although a substantial transcriptional activity was observed during the NA phase

**Figure 4:**
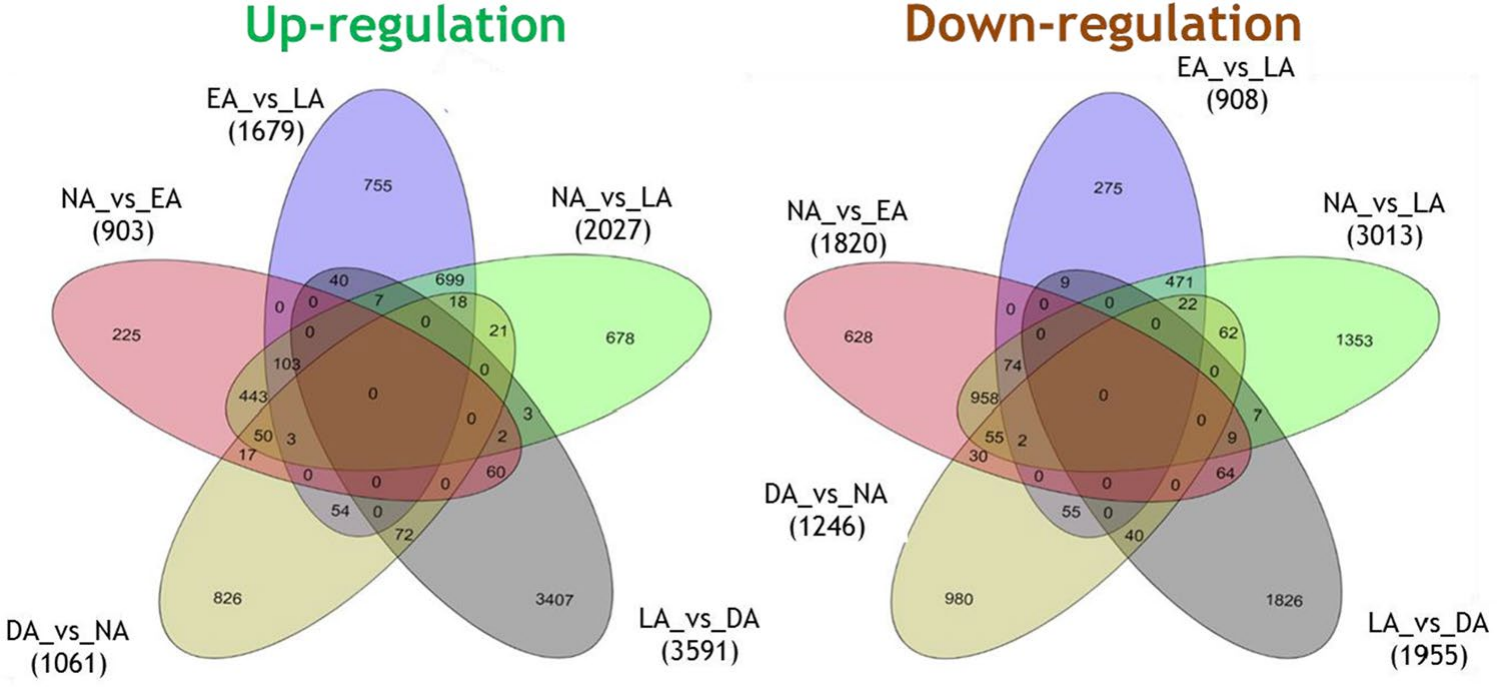
Venn diagram showing the ‘common’ and ‘specific’ differentially expressed genes [up-regulated (left) and down-regulated (right)] in pair wise comparison among the different acclimation phases. NA_vs_EA: regulation of genes during Early acclimation (EA) versus Non-acclimation (NA); EA_vs_LA: regulation of genes during Late acclimation (LA) versus EA; NA_vs_LA: regulation of genes during LA versus NA; LA_vs_DA: regulation of genes during De- acclimation (DA) versus NA and DA_vs_NA: regulation of genes during NA versus DA.

To identify the genes that were differentially expressed between consecutive phases, fold change in unigenes in a ‘given phase’ with respect to its ‘previous phase’ was calculated (Fig. 4). A total of 2723 unigenes (903 up-regulated, 1820 down-regulated) expressed differentially during the early phase of cold acclimation (i.e., NA to EA transition), whereas 2587 unigenes (1679 up-regulated and 908 down-regulated) were differentially expressed during the late phase of cold acclimation (i.e., EA to LA transition). Of these DEGs, 225 unigenes showed significant up-regulation and 628 unigenes showed down-regulation, which was unique to EA phase, whereas 755 unigenes showed up-regulation and 275 unigenes showed down-regulation unique to LA phase (Fig. 4). Overall, a total of 5040 unigenes (2027 up-regulated, 3013 down-regulated) were differentially expressed during the cold acclimation process (from NA to LA). Further, a total of 5546 unigenes (3591 up-regulated and 1955 down-regulated) were found to be differentially expressed during the transition from LA to DA phase. 3407 unigenes of these DEGs showed up-regulation, whereas 1826 unigenes showed down-regulation unique to DA (Fig. 4). Likewise, 2307 unigenes (1061 up-regulated and 1246 down-regulated) were differentially expressed during DA to NA transition, wherein 826 up-regulated and 980 down-regulated unigenes were unique to DA (Fig. 4).

The expression patterns of DEGs suggested that the down-regulation of these genes was the highest during cold acclimation. This also indicates towards slowdown of most of the cellular processes in plants as they acclimate (to cold) during winters. Many previous studies on cold acclimation have also reported similar patterns^55–57^. Moreover, during transition to LA (through EA), EA stood out as being the most transcriptionally differentiated phase in terms of ‘down-regulation of genes’ (most of the genes that had higher expression during NA were down-regulated). A study also reported similar ‘transcriptional slowdown’ in *Camellia sinensis* during cold acclimation^58^. However, up-regulation of a few genes (775) was also observed unique to the LA phase. These genes might have been induced by low temperature conditions. It is also possible that the genes with higher expression levels towards LA phase might be involved in regulating the key pathways that are critical for cold acclimation. Similar inferences were made for *Arabidopsis*, in which the cold acclimation-related genes showed maximum expression during the later stages of cold acclimation^59^.

The leaves of *R. anthopogon* starts developing in mid-May and mature in August (as no changes are observed in the size of leaves thereafter); hence, it was expected that the leaves during this period would be transcriptionally most active, both in terms of number of genes as well as their expression levels. It has been suggested that as the plant acclimatizes to cold conditions, cellular machinery slows down and as a result, leaves would be transcriptionally less active^58^. Further, when the plants pass through the harsh winters, there would be damage to leaves, because of which the molecular machinery will not be as efficient as during optimum growth conditions found in the month of ‘August’ of the subsequent year. In this study, the results suggested that DA was transcriptionally the most ‘active’ [with highest number of DEGs (5546)] and ‘differentially up-regulated’ phase [with highest number of up-regulated DEGs (3591)]. Although the levels of most of the genes expressed during DA phase was comparable to the expression level during the NA phase, yet 2307 genes were differentially expressed between the DA and NA phases. This result indicated that the re-commencement of cellular processes to resume plant growth occurs to full extent during the spring season^55^.

### Temporal cascades of genes during the transition across different phases

It is well-known that plants possess mechanisms that enable them to acclimate to low temperature and further revert from a cold-acclimated to a de-acclimated state to facilitate rapid resumption of normal cellular functions and metabolism^60^. However, little attention has been given on the variation in processes related to growth (e.g. photosynthesis) and defense mechanism (activation of stress responsive genes) that takes place in plants in response to environmental variability.

#### Genes encoding LEA proteins were up-regulated during the transition to LA phase

It was observed that with a drop in both the ambient air temperature and the photoperiod towards winter, the freezing resistance in leaves of *R. anthopogon* also increased resulting in ‘cold acclimation’. On the other hand, ‘de-acclimation’ was associated with decrease in freezing resistance during spring, as the temperature and photoperiod started rising. These observations regarding ‘seasonal acquisition of freezing resistance’ in *R. anthopogon* were supported by the underlying molecular processes. As also previously reported, the low temperatures activate several cold-inducible genes, such as those that encode LEA proteins including dehydrins. LEA proteins, also referred as hydrophilins, are a large and highly diverse family that accumulate in plants for protection against abiotic stresses, especially cold and drought, resulting in cellular dehydration^54,61^. In the transcriptome analysis, an up-regulation of eight LEA genes (e.g. LEA protein 1, 3, 13, Dc3-like, D-34-like, 2-like, 76 like and 14-A), was observed during cold acclimation, when the freezing resistance in *R. anthopogon* was also found to be increased (Supplementary Table S3). This enhanced expression of diverse ‘LEA protein encoding genes’, especially dehydrins, during cold acclimation is similar with the results from previous studies depicting their role in freezing resistance in other *Rhododendron* species^62,63^. With the down-regulation of LEA genes during de-acclimation process, a decrease in freezing resistance of this species was observed. Similar findings were reported for *Prunus persica* during the de-acclimation process^64^.

**Figure 5:**
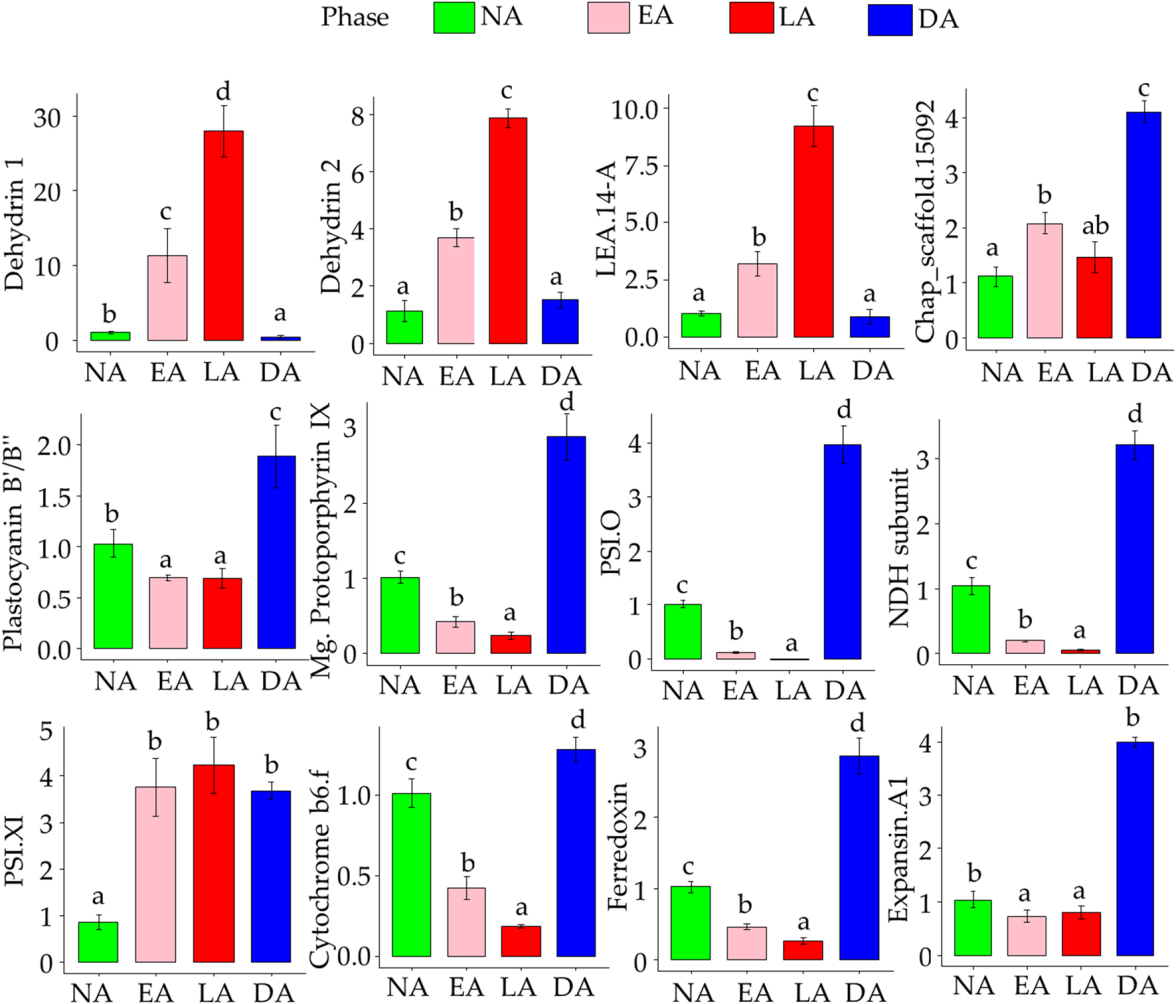
Validation of genes using RT-qPCR. Different alphabets (a, b, c, d) represent statistically significant values (*p* ≤ 0.05) as determined by Tukey’s *post-hoc* test. LEA.14A = Late Embryogenesis Abundant, Chap_scaffold.15092 = BAG family molecular chaperone regulator 7, Mg.Protoporphyrin IX = Magnesium-protoporphyrin IX monomethyl ester [oxidative] cyclase, PSI.XI = Photosystem I subunit XI and PSI.O = Photosystem I subunit O.

Dehydrins are a subset of LEA proteins, belonging to group 2 (D-11), that accumulate in response to low temperature or other desiccation triggering cellular processes in plants^65,66^. In this study, three unigenes encoding dehydrins were identified, showing differential expression between acclimation phases. It was found that the expression levels of all three dehydrins (e.g. putative dehydrin, and dehydrin 1 and 2) appeared to be highly up-regulated (~ fivefold) during cold acclimation (mainly EA), and further down-regulated during the DA phase. In addition, it was also observed that two of these dehydrin encoding unigenes (putative dehydrin and dehydrin 1) were also considerably up-regulated during NA phase, when compared to the DA phase (Supplementary Table S3). An increase in dehydrin levels has also been associated with low temperature acclimation in several other species, such as *Betula pubescens*^67^, *Pinus sylvestris*^68^*, Picea obovata*^69^ etc*.* The expression of these genes was subsequently reduced during the DA phase. The reliability of RNA-seq data was confirmed through RT-qPCR expression analysis of three genes encoding LEA and dehydrins (Fig. 5). The expression of genes encoding LEA and dehydrins were several folds higher during cold acclimation, as compared to de-acclimation and non-acclimation (Fig. 5).

Further, the expression level of four unigenes (e.g. LEA 5-D-like, LEA-14 protein, LEA and LEA protein At1g64065-like) showed up-regulation during DA phase. Moreover, the expression levels of a few LEA genes were found to be increased during the DA and NA phases. The end-products of such genes (proteins/metabolites) might have a role in combating dehydration in *R. anthopogon*, which occurs due to ‘high light conditions’ observed during spring and summer at the study site. Moreover, certain LEA genes (e.g. LEA protein Dc3, D-29, D-29-like and group 3-like) had higher expression levels during the NA phase, when compared to DA, despite similar temperature patterns observed during these two phases (i.e., 7.9 °C during DA phase and 9.4 °C during NA phase). This could be due to the fact that leaves of the studied plant species tend to remain freezing tolerant across the year to withstand any sudden ’frost events’. So, these genes might play an important role in combating dehydration stress caused due to such events at high altitudes. Overall, the diversity of LEA encoding genes might have a role in withstanding vagaries in natural conditions by adjusting their expression levels.

#### Genes encoding heat shock proteins (HSPs) were differentially expressed among the four phases

Molecular chaperones/HSPs are key components contributing to cellular homeostasis under both optimal and adverse growth conditions^70^. These play a crucial role in re-establishment of normal protein conformations^71,72^. In this study also, a large number of genes encoding HSPs were found to be differentially expressed among the four phases (Supplementary Table S3). For instance, ten HSPs members, including HSP20.1, HSP21 (a heat shock memory-related gene), stromal and mitochondrial HSP70 (3 unigenes), heat shock cognate 70 kDa protein (2 unigenes), heat shock cognate protein 80-like, HSP83-like, HSP90, HSP-90-1, HSP-90-5 and a heat shock factor (HSF; play a major role in activating the transcription of other HSPs/chaperones^72^) protein, were significantly up-regulated during EA, whereas, eight HSPs, including HSP70 (5 unigenes), small HSP, HSF protein, HSF-30-like, HSP17.1, HSP17.3, HSP20 and HSP21.4, were up-regulated during transition to LA phase. The expression level of most of these unigenes was down-regulated during further transition to DA phase. Moreover, the expression of three HSPs was found to be up-regulated during both EA and LA, which further down-regulated during transition to DA phase (e.g. HSP20-like, stromal HSP70 and small HSP). It was found that expression of three unigenes encoding chaperones was up-regulated exclusively during cold acclimation (EA or LA) [e.g. chaperone protein like and chaperone protein dnaJ 3 (2 unigenes)]. Three chaperones encoding unigenes showed increase in the expression levels during late phase of cold acclimation (i.e., LA), and down-regulation during DA [e.g. chaperone protein dnaJ C76 and ClpB1 (an Hsp100 family protein)] or NA phase (e.g. chaperone protein ClpD). These members of HSP20, HSP30, HSP70 etc. probably play a key role in cold stress tolerance. The results corroborated with the findings that have also observed a higher expression level of genes encoding HSPs during cold acclimation^73^.

Contrary to this, several members of HSF gene family were significantly up-regulated during de-acclimated state of plants (Supplementary Table S3). For example, four unigenes encoding chaperones exhibited up-regulation during DA phase exclusively [e.g. chaperone protein like, dnaJ 11 (2 unigenes) dnaJ 11 like]. In comparison to cold acclimation, expression levels of seven members of HSPs, including HSP9/12, HSP12 (2 unigenes), HSP18.2 (a heat shock memory-related gene), HSP21.7, HSP90-6, HSP-SSB and HSF-B3, were significantly up-regulated during DA phase. Similarly, a chaperone encoding unigene was found to be up-regulated during DA phase (i.e., BAG family molecular chaperone regulator 7). Further, the expression of a unigene (i.e., copper chaperone) appeared to be up-regulated during DA as well as NA phase, when compared to cold acclimation. Three unigenes [ATP-dependent chaperone ClpB (an Hsp100 family protein)], Chaperone protein dnaJ 8 and dnaJ 11 like) showed an increase in the expression levels during DA phase, but down-regulated during NA. In contrast, the expression of three unigenes encoding HSPs was found to be significantly up-regulated [e.g. HSP70, HSP82 (~ sevenfold) and perikaryon HSP (~ 13 fold)] during NA phase when compared to DA phase (Supplementary Table S3).

The temperatures such as 15–20 °C could not normally be presumed as a heat stress, as similar temperature could be observed during NA conditions. However, the up-shift in temperature by ~ 30 °C [from ‘sub-zero temperatures (− 15 °C) during winter’ to ‘15 °C during summer’] as observed in this alpine site, might have apparently been perceived as heat stress by the plants. The HSP genes expressed during DA phase might play a key role in providing tolerance to this temperature increase, observed during this phase. The higher expression levels of genes encoding HSPs could also be high due to high albedo effect and high radiations during DA phase. A study on *Deschampsia antarctica* demonstrated that the ‘cold-acclimated plants (4 °C)’ when subjected to thermal stress (35 °C) accumulated more HSP proteins in their leaf tissue than the ‘control plants (13 °C)’^74^. Hence, this class of genes might be involved to reset the protein conformations during the DA phase.

Overall, the results showed that genes encoding for HSPs and their regulator HSFs showed a differential expression throughout the year, which suggest their seasonal variations-specific roles. HSPs play indispensable roles during growth and development as well as during environmental perturbations; however, there lies a specificity towards the cue^70,71^. For instance, specific HSPs such as HSP90 and HSP70 proteins, together with chaperonins, are involved in post-translational protein folding and targeting linked to primary metabolism. Up-regulation of respective HSPs during DA and NA phases, is indicative of active metabolism. On the contrary, larger HSPs such as ClpB and ClpD and small HSPs are implicated with stress conditions where they either protect the proteins from misfolding or facilitate the degradation of mis-folded and damaged proteins to maintain cellular homeostasis. Up-regulation of such HSP and cognate HSFs during EA and LA phases reveals their canonical roles in protecting plants from stress and maintaining protein homeostasis, allowing plants to withstand damaging freezing stress^71–73^.

#### Genes related to photosynthetic machinery are maximally up-regulated during NA and DA, whereas these are down-regulated during LA phase

Photosynthesis is a key physiological process underlying plant growth and development. The harsh environmental conditions, especially the low temperatures, are known to significantly slow down this process^75^. In this study also, the majority of photosynthetic-enzyme related genes associated with light reactions (e.g. PS-I and PS-II, photophosphorylation, antenna proteins etc.), along with genes pertaining to chlorophyll biosynthesis were down-regulated during the LA phase (Supplementary Table S4). In addition, the enzymatic reactions of the Calvin-Benson cycle were found to be slowed down during low temperature conditions (the lower expression of Calvin cycle related genes observed during LA phase). Though, the expression of certain genes (e.g. PS-I reaction center subunit N, III and VI-1, oxygen-evolving enhancer protein 1 and 2, chlorophyll a-b binding protein 3C, 5, CP29.2, CAB11 etc.) was found to be up-regulated during EA, but a substantial decrease in expression was observed during transition to LA phase (Supplementary Table S4). The decline in expression of genes related to photosynthetic machinery under cold conditions was supported by the fact that the photosynthetic rates and stomatal conductance during the cold acclimation process were also observed to be low (Fig. 2, Tables 2, 3). In RT-qPCR expression analysis of genes related to photosynthetic pathway and porphyrin metabolism, the lowest expression was recorded during cold acclimation (Fig. 5). The transcriptome data along with field measurements, supported the hypothesis that photosynthetic machinery gets impaired on exposure to low temperatures in *R. anthopogon*. These findings were in agreement with earlier studies, which demonstrated inhibition of photosynthesis during cold stress^75–78^. Previous studies have reported that a decrease in temperature results in cessation of growth, which greatly reduces the carbon sink capacity in leaf tissues. The decrease in carbon sink consequently reduces rate of cellular respiration and induces negative feedback regulation of carbon assimilation^79^. For compensating reduced energy sink, plants reduce their capacity for harvesting sunlight to maintain photostasis. This is achieved by adjusting photosynthetic pigments, and by down-regulating the expression of genes related to light reaction^80^.

Considering the observed annual air temperature profile of the study site, it was expected that NA phase would be the transcriptionally most active, with photosynthetic machinery maximally up-regulated in this phase. However, in contrast, DA phase was found to be the ‘photosynthetically’ most active phase, as indicated by higher enrichment of genes involved in photosynthesis (Supplementary Table S4). These DEGs covered all the segments of photosynthetic machinery, including components of both photosystems (PS) i.e., PS-I (e.g. PS-I subunit O, PS-I reaction center subunit IV B and VI, nifU-like protein 2 etc.) and PS-II (e.g. PS-II oxygen-evolving complex protein 2), photophosphorylation electron transport (e.g. cytochrome b6/f complex, plastocyanin, ferredoxin, psbQ-like protein 3), F-type ATPase (e.g. ATP synthase CF0 subunit I protein), enhancers (e.g. oxygen-evolving enhancer protein like) and photo-damaged repair of PS (e.g. psbP-like protein 1). These results corroborated with the findings of another study, which suggested an up-regulation of genes related to photosynthesis in *Arabidopsis thaliana* during de-acclimation^59^. Also, the expression level of genes associated with ‘photosynthesis-antenna proteins’ (e.g. chlorophyll a-b binding protein 3, 3-1, P4, 5, 6A, 13A etc.) and ‘porphyrin and chlorophyll metabolism’ (e.g. tripartite terminase, magnesium-protoporphyrin IX monomethyl ester cyclase, magnesium-chelatase subunit ChlH like, light-harvesting complex-like protein OHP1 etc.), was enhanced during the DA phase, which was higher than that observed during NA. In RT-qPCR expression analysis of genes related to photosynthetic pathway and porphyrin metabolism, the highest expression was recorded during de-acclimation, followed by non-acclimated state (Fig. 5). The expression of a gene, PSI.XI, was up-regulated during transition to EA from NA, but did not change further during LA and DA phases. PSI.XI plays a role in stabilizing the light harvesting complexes (Lhca) 2 and 3^81^, and therefore, its increase can be attributed to stabilize these complexes during stressful conditions. Similar findings were reported in *Arabidopsis thaliana* with clear evidence of a transition to growth and development at transcript levels during the de-acclimation period^15^. In this study, it was also found that there was an up-regulation of genes encoding several families of ‘transcription factors’ controlling plant development during de-acclimation. A similar study on transcriptome analysis in *Arabidopsis* leaves during cold acclimation, de-acclimation and re-acclimation revealed that the genes related to photosynthetic machinery were up-regulated more during the de-acclimation phase, when compared to other phases^55^. This suggested that *R. anthopogon* assimilates carbon (by activating the photosynthetic machinery) during both during summer (represented by NA phase) as well as spring (represented by DA phase).

Further, the results also revealed that PS-I activity was more enhanced relative to PS-II during the DA phase, as genes related to PS-I were over-represented in photosynthetic DEGs during the transition from cold-acclimated to de-acclimated state. This suggested that ‘cyclic electron transport’ was enhanced in *R. anthopogon* to support the ‘linear electron transport’. The increase in cyclic phosphorylation would provide an alternative electron sink under the ‘high light’ and ‘cold’ conditions observed during the DA phase. It has been previously reported that high light intensity creates surplus electrons from the photosynthetic light reactions relative to the electron-accepting capacity of stroma. This eventually leads to ‘acceptor side’ limitation at PS-I^82^. Therefore, the observed increase in expression of PS-I related genes could be to avoid this ‘acceptor side’ limitation by increasing the abundance of PS-I in leaf tissues. However, it is also possible that due to ‘high solar radiation intensity’ and ‘albedo’ effect prevalent during the DA phase, PS-I gets damaged and high transcription of PS-I related genes occurs to replace the damaged proteins. Further, the increased expression of a gene encoding psbP protein suggested that photochemistry was strengthened through acceleration of repair of the PS-II reaction-centre core proteins, possibly damaged due to photo-inhibition^83^. In addition, it was found that the expression level of genes related to ‘carbon fixation’ (e.g. those encoding for glyceraldehyde-3-phosphate dehydrogenase, sedoheptulose-1;7-bisphosphatase etc.) was also up-regulated during the DA phase, suggesting an increase in the regeneration of ribulose bisphosphate (RuBP), thereby increasing the efficiency of Rubisco carboxylation (i.e., carbon fixation).

Overall, the observed gene expression patterns suggest that there was an increase in ‘PSI reaction centre’, ‘chlorophyll pigments’ and ‘photosynthetic carbon fixation’ (i.e., Calvin cycle) in leaves of *R. anthopogon* during the transition to DA phase. The transcriptome analysis also revealed increased levels of genes, which expressed in ‘response to light’ (e.g. expansins), during de-acclimation. These genes are known to be involved in cell elongation process. Similar findings regarding leaf expansion were observed during de-acclimation in *Brassica napus*^84^. All these changes in molecular machinery could be important to meet the requirements of plants to synthesize new tissues during the DA phase.

## Conclusion

Our time-series eco-transcriptomics study provided important insights into the transcriptome dynamics during the annual growth cycle of an evergreen shrub occurring at high elevation environments of Himalaya. Our results revealed that the leaves undergo reshuffling of the transcriptome in a phase-specific and directional manner (the expression of most of the genes increase towards winters and decline during spring). Further, the transcriptome changes were maximum during the transition to de-acclimation compared to cold acclimation, indicating it to be the transcriptionally most active phase. It was observed that de-acclimation was not just a reversal of cold acclimation but also involved specific changes implicated in growth and development. Overall, the findings suggest that the plants in varying environments, such as high altitudes, remodel and reconfigure their transcriptome to sustain the climatic variability.

## Supplementary Information

Supplementary Information.

## Data Availability

The dataset generated during the current study have been deposited in NCBI's BioProject Accession Number PRJNA751462.
