# Supplementary material for: Time-series RNA-Seq transcriptome profiling reveals novel insights about cold acclimation and de-acclimation processes in an evergreen shrub of high altitude
Source: Sci Rep. 2022 Sep 16;12:15553. doi: 10.1038/s41598-022-19834-w (PMC9481616; doi:10.1038/s41598-022-19834-w)
Supplement: Supplementary file 1 — Supplementary Information. [file 41598_2022_19834_MOESM1_ESM.docx]

**SUPPORTING INFORMATION**

**Time-series RNA-Seq transcriptome profiling reveals novel insights about cold acclimation and de-acclimation processes** **in an evergreen shrub of high altitude**

Nikita Rathore^1,3#^, Prakash Kumar^2,3,4#^, Nandita Mehta^1,3^, Mohit Kumar Swarnkar^2^, Ravi Shankar^2,3,4*^ and Amit Chawla^1,3*^

Corresponding authors: [amitchawla21@gmail.com](mailto:amitchawla21@gmail.com) and [ravish@ihbt.res.in](mailto:ravish@ihbt.res.in)

^1^Environmental Technology Division, CSIR-Institute of Himalayan Bioresource Technology (CSIR-IHBT), Palampur, H.P, India;

^2^Biotechnology Division, CSIR-IHBT, Palampur, H.P, India;

^3^Academy of Scientiﬁc and Innovative Research (AcSIR), Ghaziabad- 201002, India.

^4^Studio of Computational Biology & Bioinformatics, The Himalayan Centre for High-throughput Computational Biology (HiCHiCoB, A BIC of Department of Biotechnology, Govt. of India), CSIR-IHBT, Palampur, H.P, India

^#^Contributed equally to this work.

**Funding source:** Council of Scientific and Innovative Research (CSIR), Government of India, under the research grant MLP-0145.

**METHODS**

***De novo assembly***

After quality assessment using Trimmomatic**^1^**, *de novo* assembly of high quality reads was performed using SOAP *de novo*-Trans-127mer tool, a de Bruijn graph-based assembler**^2^**. The reads were first split into smaller pieces, the ‘k-mers’, for assembly in order to produce contigs. Varying ‘k-mer’ lengths between 21 to 99 and average insert size of 300 bp of PE reads were used to screen out the best performing assembly. The *de novo* assembly constructed with a ‘*k*-mer’ size set as 91 achieved the best balance between the number of contigs produced, coverage and average sequence length attained. Similar information was also used to build the scaffold sequences by merging two contigs into single scaffold sequence, sharing the read-pairs. The final assembled transcriptome comprising of 74,769 contigs was additionally improved using three ‘clustering based’ approaches; first, an ‘overlap-based clustering’ using TGICL-CAP3 was performed with terminal overlap of 40 bp and 90 % identity for terminal joining which retrieved 46,736 contigs (merged as well as singleton), second, for removing redundancy between the contigs (retrieved from overlap based clustering), ‘Cluster database at high identity with tolerance (CD-HIT-EST) clustering’ was performed with 95 % similarity cut-off, yielding a total of 43,295 contigs, and third, a ‘dissimilar sequence (DS) clustering’ approach was implemented to further cluster contigs which did not have any sequence similarity but belonged to different regions of a gene. The DS clustering retrieved a total of 32,142 unigenes. Further, the unigenes having ‘fragments per kilobase of transcript per million’ (FPKM) value > 0 in at least two replications of any given sample (time-point) were designated as expressed genes, and finalized for use in subsequent analysis. Thus, a final set of 29,096 unigenes were obtained that have expressed at least during a single time-point.

***Functional annotation and classification***

In order to identify and assign biological functions to each gene, the assembled transcripts were searched against NCBI’ non-redundant protein (NR) and UniProt databases using BLASTX with an e-value threshold of 1.0e^-5^, as described in earlier studies**^3,4^**. Further, gene ontology (GO) terms were assigned for each unigene based on GO category of its best-aligned significant hit (with highest bit score and sequence identity) in UniProt database. Kyoto Encyclopedia of Genes and Genomes (KEGG)**^5^** and Enzyme Commission (EC) annotation and classification of the assembled transcripts was done using a similar approach.

**References**

1. Bolger, A.M., Lohse, M., & Usadel, B., 2014. Trimmomatic: a flexible trimmer for Illumina sequence data. Bioinformatics, 30, 2114-2120. https://doi.org/10.1093/bioinformatics/btu170
2. Xie, Y., Wu, G., Tang, J., Luo, R., Patterson, J., Liu, S., Huang, W., He, G., Gu, S., Li, S. and Zhou, X., 2014. SOAPdenovo-Trans: de novo transcriptome assembly with short RNA-Seq reads. Bioinformatics, 30, 1660-1666. https://doi.org/10.1093/bioinformatics/btu077
3. Dhiman, N., Sharma, N.K., Thapa, P., Sharma, I., Kumar Swarnkar, M., Chawla, A., Shankar, R. and Bhattacharya, A., 2019. De novo transcriptome provides insights into the growth behaviour and resveratrol and trans-stilbenes biosynthesis in *Dactylorhiza hatagirea*-An endangered alpine terrestrial orchid of western Himalaya. Scientific reports, 9, 1-13. https://doi.org/10.1038/s41598-019-49446-w
4. Gahlan, P., Singh, H.R., Shankar, R., Sharma, N., Kumari, A., Chawla, V., Ahuja, P.S. and Kumar, S., 2012. De novo sequencing and characterization of *Picrorhiza kurrooa* transcriptome at two temperatures showed major transcriptome adjustments. BMC genomics, 13, 1-21. https://doi.org/10.1186/1471-2164-13-126
5. Kanehisa, M., Goto, S., Kawashima, S., Okuno, Y. and Hattori, M., 2004. The KEGG resource for deciphering the genome. Nucleic acids research, 32, 277-280. https://doi.org/10.1093/nar/gkh063

**RESULTS**

***Key characteristics of de novo sequence assembly***

A total of 36 RNA-seq libraries representing 12 different time-points (n=3 for each) were subjected to paired-end (PE) sequencing on Illumina Novaseq platform. The PE sequencing resulted into a total of 718.2 million PE reads, ranging from 11 to 27.8 million reads for each library. *De novo* assembly of high-quality reads (>Q30 score) generated a total of 74,769 transcripts (at 91-kmer), with an average length of 664 bp and N50 value of 1448 bp. The TGICL-CAP3 clustering approach further reduced the transcript number from 74,769 to 46,736 transcripts. Further, CD-HIT clustering retrieved 43,295 transcripts. Out of these 43,295, significant BLAST hit in NCBI’s Nr database were found for 38,806 (89.6 %) transcripts. On the basis of DS clustering approach, the transcripts were further merged, and this reduced the transcript number to 32,142. After count filtering among biological replicates, a final set of 29,096 unigenes were obtained, which were further subjected to differential gene expression analysis.

***Functional annotation of assembled transcripts***

Similarity search of sequences against different species revealed a maximum homology with *Actinidia chinensis* (36.4 %), followed by *Camellia sinensis* (30.2 %) and *Nyssa sinensis* (8.8 %). The top 20 plant species showing homology with *R. anthopogon* in blast similarity search are shown in Supplementary Figure S3.

To gain an insight on probable functions of transcripts, assembly was subjected to annotation analysis. Primarily, all the assembled transcripts of *R. anthopogon* (43,295; from CD-HIT clustering) were used in a “BLASTX search” against the NCBI’s Nr database (weblink-ftp://ftp.ncbi.nlm.nih.gov/blast/db/). The query was considered to have a possible homolog in a database for e-values ≤ l.0e-5. A total of 38,806 transcripts (89.7 %) had significant hit to genes showing putative functions. The transcripts having significant hit in Nr database were further characterized for “GO terms” in biological process, cellular component and molecular functions. A total of 27,007 transcripts were successfully assigned to 8,505 GO terms, of which 4,819 (56.7 %) were categorized into biological process, 1038 (12.2 %) into cellular component and 2,648 (31.1 %) were classified into molecular function. In ‘biological process’ category, response to abscisic acid stimulus process (1 %) was the highly represented group followed by regulation of transcription (0.85 %) and embryo development ending in seed dormancy (0.77 %). The classification in ‘cellular component’ category revealed that the maximum number of transcripts localized to nucleus (10.4 %), followed by cytosol (8 %) and plasma membrane (6.8 %). In ‘molecular function’ category, protein binding (22.2 %), RNA binding (2.8 %) and sequence-specific DNA binding transcription factor activity (2.2 %) were the most enriched GO terms. The top 20 GO enriched terms (associated with the annotated transcripts) in various categories (biological processes, cellular components and molecular functions) are presented in Supplementary Figure S4.

To elucidate active biosynthesis pathways operative in *R. anthopogon*, annotation with KEGG database could be obtained for 15,614 assembled transcripts, comprising of 269 unique KEGG orthology identifiers. The KEGG pathway analysis showed a predominance of plant hormone signal transduction pathway (5.8 % transcripts), followed by plant-pathogen interaction (5.1 %), protein processing in endoplasmic reticulum (3.6 %), ribosome (3.5 %) and spliceosome (3.3 %). The sequence homology search against enzyme classification categories revealed non-specific serine/threonine protein kinase as the most abundant class.

***GO and KEGG pathway enrichment of DEGs***

GO enrichment analysis of DEG’s was carried out to summarize the key GO categories enriched during the different acclimation phases (p ≤ 0.05). Among the enriched biological processes, genes with annotations of ‘multi-organism process’ (GO: 0051704), ‘response to stimulus’ (GO: 0050896) (biotic, abiotic and chemical stimulus, and response to stress) and ‘cell wall biogenesis and modification’ (GO: 007155) were found to be enriched during the EA phase (*i.e.,* the transition from NA to EA). However, no GO enrichment was found during the LA phase (*i.e.,* from EA to LA transition). The DA phase (*i.e.,* LA to DA transition) was found to be enriched for ‘multi-organism process’ (GO: 0051704), ‘response to stimulus’ (GO: 0050896), ‘cell wall organization or biogenesis’ (GO: 007155) and ‘secondary metabolic process’ (GO: 0019748). On the other hand, the genes with annotations of ‘cell wall biogenesis and modification’ (GO: 007155), ‘cellular amino acid derivative metabolic process’ (GO: 0006575) and ‘secondary metabolic process’ (GO: 0019748) were enriched during the NA phase (*i.e.,* DA to NA transition).

In ‘molecular function’ category, genes with annotations of ‘catalytic’ (GO: 0003824) and ‘transporter’ activity (GO: 0005215) were highly enriched during the EA phase (*i.e.,* NA to EA transition), whereas, genes with annotation of ‘fatty acid elongase activity’ (GO: 0009922) were found to be enriched during the LA phase (*i.e.,* EA to LA transition). The DA phase (*i.e.,* LA to DA transition) was found to be enriched for GO terms related to ‘transporter activity’ (GO: 0005215) (substrate-specific and transmembrane transporter activity), ‘catalytic activity’ (GO: 0003824) (oxidoreductase activity), ‘molecular transducer activity’ (GO: 0060089) and ‘peptide binding’ (GO: 0042277). The GO terms associated with ‘transporter’ (GO: 0005215) and ‘catalytic’ activity (GO: 0003824) (oxidoreductase and transferase activity) were enriched during the NA phase, when compared to the DA phase.

In the ‘cellular component’ category, genes with annotations of ‘cell wall’ (GO: 0005618), ‘plasma membrane’ (GO: 0043229) and ‘apoplast’ (GO: 0048046) were found to be enriched during the EA phase (*i.e.,* NA to EA transition), whereas, LA phase (*i.e.,* EA to LA transition) was enriched for GO terms associated with ‘apoplast’ (GO: 0048046) and ‘cytoplasmic mRNA processing body’ (GO: 0000932). The DA phase (*i.e.,* LA to DA transition) was found to be enriched for GO terms related to ‘apoplast’ (GO: 0048046), ‘symplast’ (GO: 0000121), ‘external encapsulating structure’ (GO: 0050896) (cell wall, casparian strip), ‘membrane’ (GO: 0016020) (plasma membrane, thylakoid membrane *etc.*) and ‘vacuole’ (GO: 0005773) (plant type, vacuolar membrane). Similarly, the NA phase, when compared to DA phase (*i.e.,* the transition from DA to NA), was enriched for GO terms associated with cell wall (GO: 0005618), cell membrane (GO: 0016020) and the vacuole (GO: 0005773) (plant type vacuole).

Similarly, KEGG enrichment analysis of DEG’s was carried out to determine the key pathways enriched during the different acclimation phases (p ≤ 0.05) (Supplementary Table S2). It was found that the genes with KEGG annotation of ‘pentose and glucuronate metabolism’, ‘plant hormone signal transduction pathway’ and ‘plant-pathogen interaction’ were found to be significantly enriched during EA (*i.e.,* NA to EA transition), DA (*i.e.,* LA to DA transition) and NA phase (DA to NA transition). LA phase (*i.e.,* EA to LA transition) was exception, wherein the genes with KEGG annotation of ‘plant-pathogen interaction’, ‘protein processing in endoplasmic reticulum’ and ‘phenylpropanoid biosynthesis’ were found to be significantly enriched.

**SUPPLEMENTARY FIGURES**


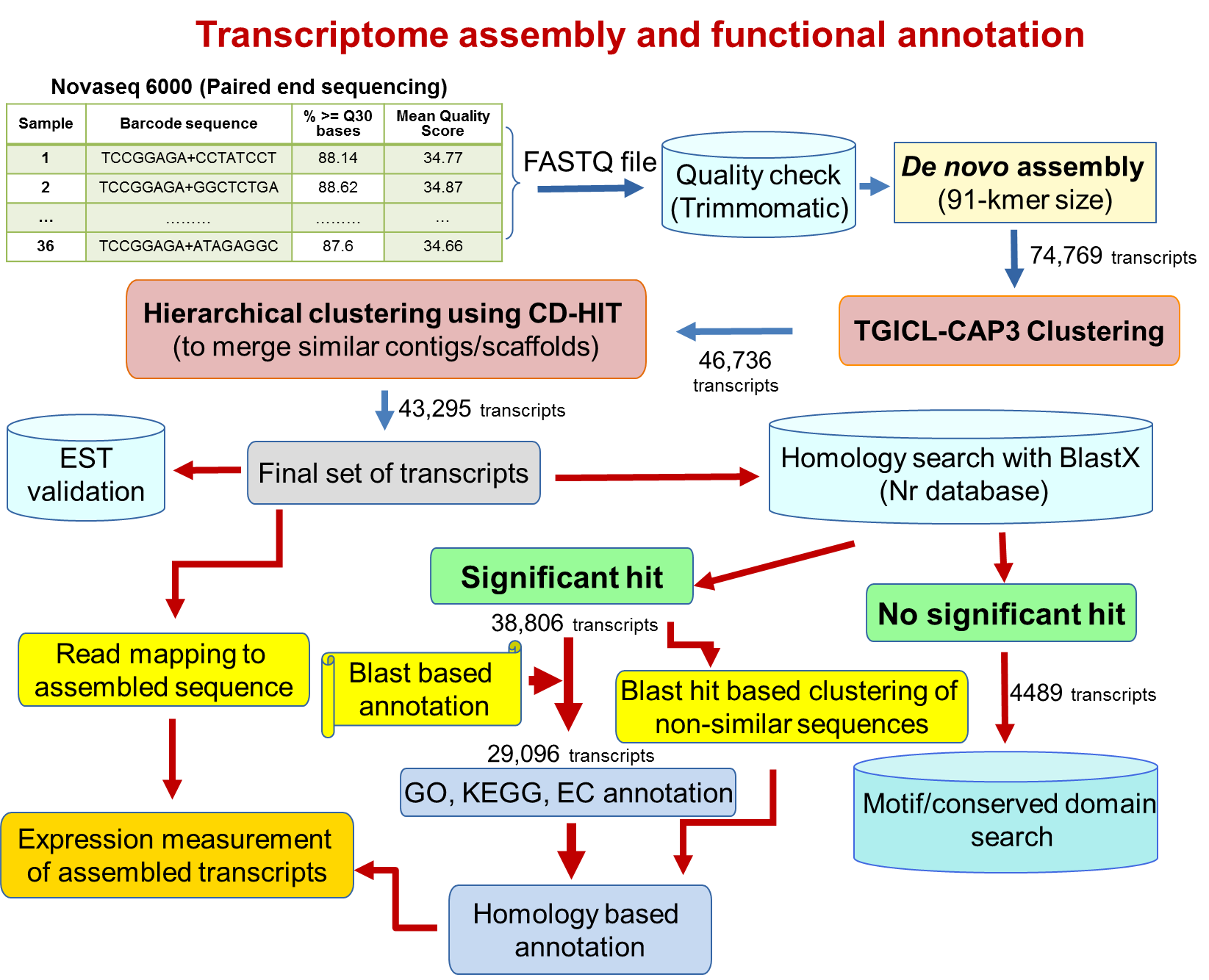


**Supplementary Figure S1.** Steps followed during *de novo* transcriptome assembly and functional annotation of *R. anthopogon*.

***
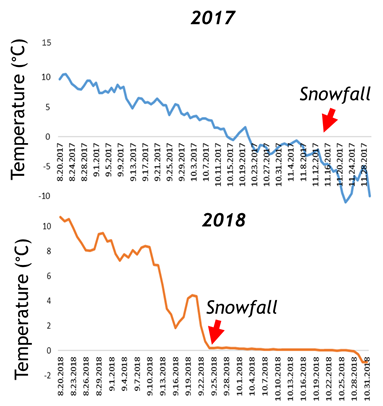
***

**Supplementary Figure S2.** Graphs showing change in daily mean air temperature (°C) at the study site for two consecutive years [*i.e.,* 2017 (upper) and 2018 (lower)] during the time of snowfall. Red arrows point to the day of snowfall after which plants got covered at the study site under the snow until next June. The temperature patterns at the time of snowfall in 2017 (*i.e.,* 14-November-2017) were similar (*i.e.,* <0 °C) to those in 2018 (*i.e.,* 24-September-2018).


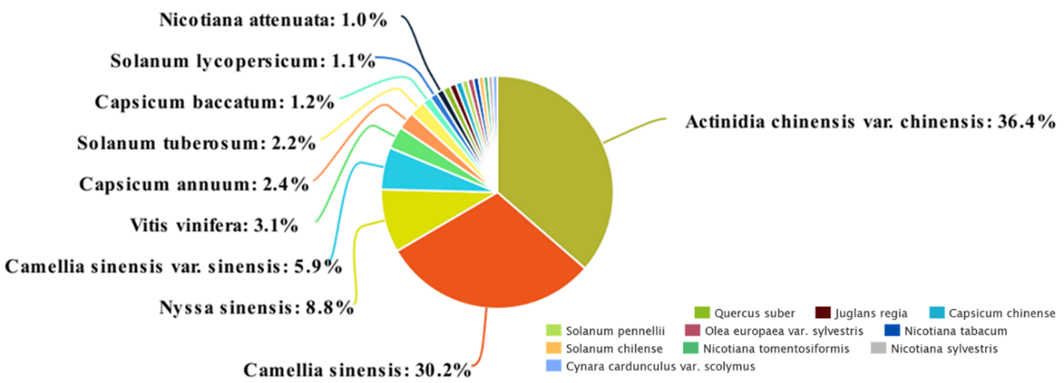


**Supplementary Figure S3.** Species homology with *R. anthopogon* in BLAST similarity search.

**
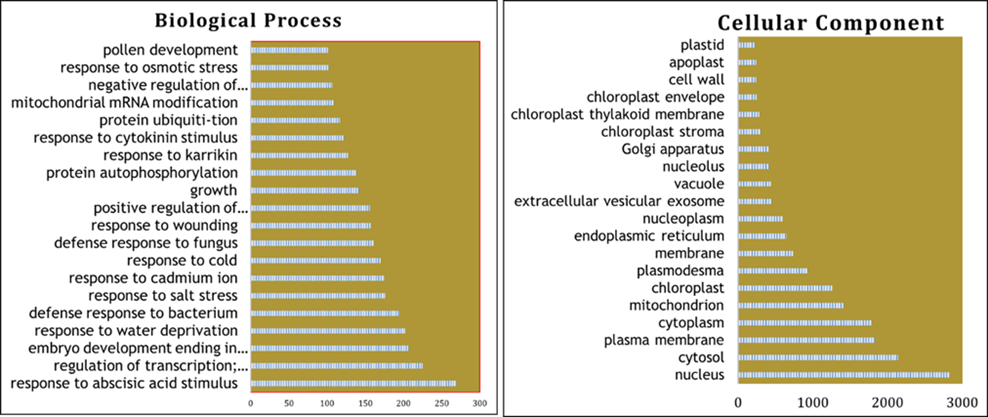
**


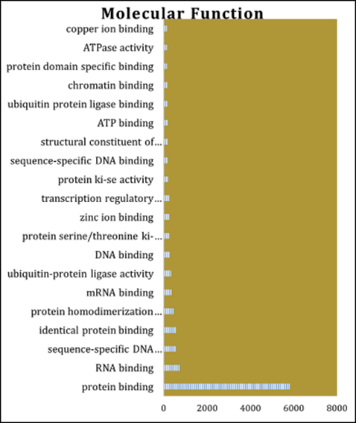


**Supplementary Figure S4.** Top 20 GO terms (*i.e.,* biological processes, cellular components and molecular functions) for genes showing significant hit in annotation analysis.


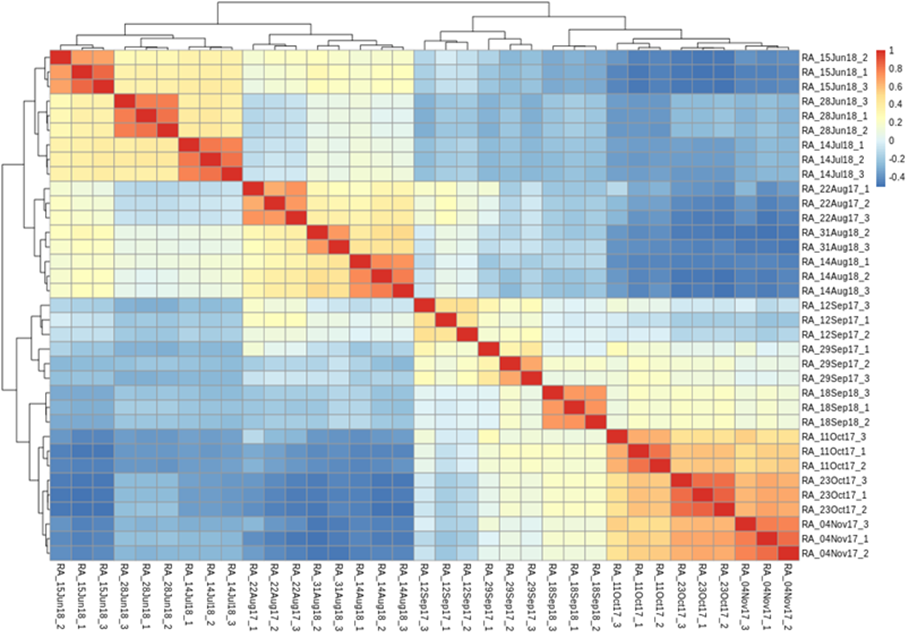


**Supplementary Figure S5.** Similarity of pattern of change in gene expression levels across sampling time-points measured using “Pearson correlation analysis” [a correlation coefficient based similarity measure for clustering time-course gene expression data (Son and Baek, 2008)]. The Pearson correlation coefficient value around one (shown in red colour) correspond to highly correlated “time-point” pairs, whereas values near zero (shown in blue colour) correspond to uncorrelated time-points.

**
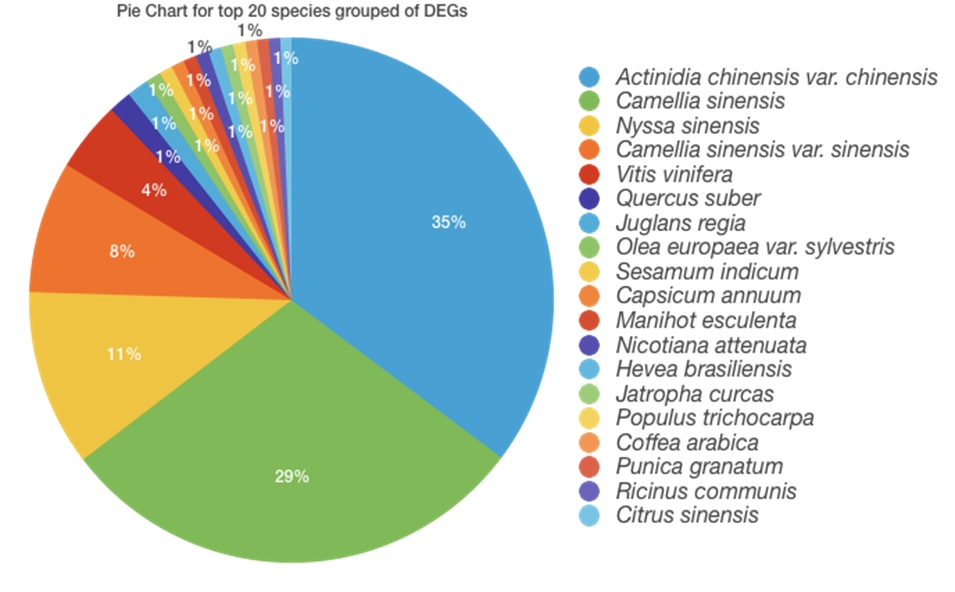
**

**Supplementary Figure S6.** Species homology (top 20) of significant differentially expressed genes.


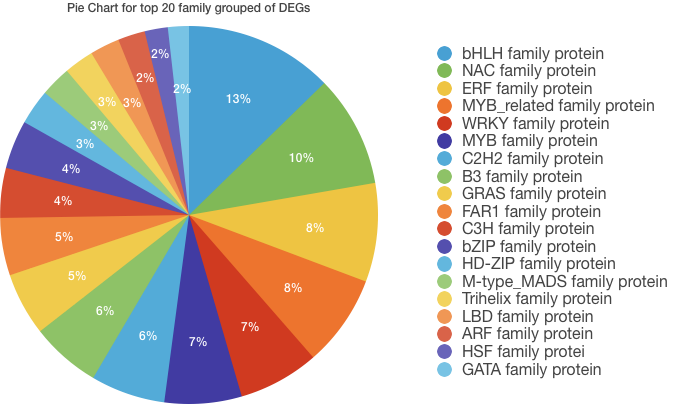


**Supplementary Figure S7.** Protein family classification (top 20) of significant differentially expressed genes.

**SUPPLEMENTARY** **TABLES**

**Supplementary** **Table S1.** DEG-specific primers for validation using real-time quantitative PCR.

| **S.no** | **Contig_ID** | **Gene Annotation** | **Forward and Reverse primer** |
| --- | --- | --- | --- |
| 1 | CL4491Contig1 | Plastocyanin B'/B'' | TCGGCAATCCAACAGCAAT  CGAGCCATCCGTTCAGTAATC |
| 2 | CL476Contig2 | Magnesium-protoporphyrin IX monomethyl ester [oxidative] cyclase | GGGAGGGCTCTGTTCTATTGG  ACATCCAGGCCCTCCATTG |
| 3 | C153656 | Photosystem I subunit O | CAATTTGCCCGAAGGTAAGG  CCACTCCCCCTGCTCTCA |
| 4 | C164326 | Photosynthetic NDH subunit | GGTGGATTGCCTGGAACATT  TGGTGGCAGTGGCTGTATGA |
| 5 | CL867Contig1 | Photosystem I reaction center subunit XI; chloroplastic | TTTCCTAGCCCTAAAATCTTGCA  CTGCCATCTTGCCCTTTTCT |
| 6 | CL2310Contig1 | Cytochrome b6-f complex iron-sulfur subunit 1 like | GTGCCAGGCCACCAGTATTG  CGCCCAAAAGAAGCAAATTC |
| 7 | CL584Contig1 | Ferredoxin | ATCACCCTGAGGATAAGCAACAC  AAGGTGGACCAGTCCGATCA |
| 8 | C169162 | Expansin-A1 | CAGGCTGGGAGAGGTCAAAG  GTCACCGCCACCAATTTCTG |
| 9 | C136590 | Dehydrin 1 | GGATGATGGACAAGGTGAAGGA  GCGTACATAGCGGTGGAAAAG |
| 10 | CL257Contig3 | Dehydrin 2 | CTGCTACTGGACTTCGTCAACAA  GAGGTGATCATCGAATTTGAAAAA |
| 11 | CL888Contig2 | Late embryogenesis abundant protein Lea14-A | CCAACCTGGTGGCGAACTT  GCCAAGGTTGCGGTTAAGAA |
| 12 | scaffold15092 | BAG family molecular chaperone regulator 7 | GTATCACCAACGCCGCATCT  TTCGTTTTGAGGCAGGCTTT |

**Supplementary** **Table S2.** KEGG enrichment analysis among different phases (p ≤ 0.05).

| **KEGG term** | **Number in input list** | **Number in BG/Ref** | **P value** |
| --- | --- | --- | --- |
| **Non-acclimation -> Early acclimation** | | | |
| **Photosynthesis - antenna proteins** | 16 | 40 | 5.80E-08 |
| **Plant-pathogen interaction** | 90 | 603 | 2.00E-07 |
| **Pentose and glucuronate interconversions** | 101 | 764 | 9.40E-06 |
| **Methane metabolism** | 13 | 48 | 0.00012 |
| **Phenylpropanoid biosynthesis** | 45 | 326 | 0.00084 |
| Flavone and flavonol biosynthesis | 10 | 38 | 0.00093 |
| Glucosinolate biosynthesis | 23 | 139 | 0.0014 |
| Cyanoamino acid metabolism | 29 | 195 | 0.0021 |
| Starch and sucrose metabolism | 35 | 251 | 0.0024 |
| alpha-Linolenic acid metabolism | 9 | 39 | 0.0043 |
| Biosynthesis of unsaturated fatty acids | 9 | 39 | 0.0043 |
| Plant hormone signal transduction | 78 | 682 | 0.0045 |
| Zeatin biosynthesis | 9 | 45 | 0.011 |
| Nitrogen metabolism | 14 | 86 | 0.012 |
| Inositol phosphate metabolism | 20 | 139 | 0.013 |
| Aminobenzoate degradation | 13 | 82 | 0.019 |
| Protein digestion and absorption | 11 | 68 | 0.026 |
| Brassinosteroid biosynthesis | 5 | 23 | 0.039 |
| Fructose and mannose metabolism | 30 | 258 | 0.046 |
| **Early acclimation -> Late acclimation** | | | |
| **Cyanoamino acid metabolism** | 33 | 195 | 8.10E-07 |
| **Plant-pathogen interaction** | 66 | 603 | 7.30E-05 |
| **Cysteine and methionine metabolism** | 16 | 99 | 0.0008 |
| **Photosynthesis - antenna proteins** | 9 | 40 | 0.001 |
| **Protein processing in endoplasmic reticulum** | 46 | 445 | 0.0023 |
| Glyoxylate and dicarboxylate metabolism | 10 | 57 | 0.0039 |
| Staphylococcus aureus infection | 46 | 463 | 0.0047 |
| Aminobenzoate degradation | 12 | 82 | 0.0076 |
| Galactose metabolism | 9 | 57 | 0.012 |
| Starch and sucrose metabolism | 26 | 251 | 0.017 |
| Insulin signaling pathway | 5 | 25 | 0.022 |
| Circadian rhythm - plant | 22 | 224 | 0.042 |
| Phenylpropanoid biosynthesis | 30 | 326 | 0.044 |
| **Non-acclimation -> Late acclimation** | | | |
| **Pentose and glucuronate interconversions** | 202 | 764 | 3.20E-13 |
| **Plant hormone signal transduction** | 170 | 682 | 1.90E-09 |
| **Phenylpropanoid biosynthesis** | 92 | 326 | 1.70E-08 |
| **Plant-pathogen interaction** | 147 | 603 | 8.10E-08 |
| **Photosynthesis - antenna proteins** | 19 | 40 | 2.80E-06 |
| alpha-Linolenic acid metabolism | 18 | 39 | 8.40E-06 |
| Starch and sucrose metabolism | 67 | 251 | 9.50E-06 |
| Cyanoamino acid metabolism | 54 | 195 | 2.10E-05 |
| Aminobenzoate degradation | 28 | 82 | 3.80E-05 |
| Flavone and flavonol biosynthesis | 16 | 38 | 0.0001 |
| Biosynthesis of unsaturated fatty acids | 16 | 39 | 0.00015 |
| Glucosinolate biosynthesis | 39 | 139 | 0.0002 |
| Fructose and mannose metabolism | 63 | 258 | 0.00027 |
| Protein digestion and absorption | 22 | 68 | 0.00057 |
| Other glycan degradation | 6 | 10 | 0.0018 |
| Methane metabolism | 16 | 48 | 0.0022 |
| Galactose metabolism | 18 | 57 | 0.0023 |
| Zeatin biosynthesis | 15 | 45 | 0.0029 |
| Photosynthesis | 34 | 136 | 0.004 |
| Fatty acid biosynthesis | 14 | 46 | 0.0097 |
| Glycerolipid metabolism | 50 | 228 | 0.0099 |
| Glyoxylate and dicarboxylate metabolism | 16 | 57 | 0.014 |
| Arachidonic acid metabolism | 12 | 40 | 0.018 |
| Chlorocyclohexane and chlorobenzene degradation | 11 | 37 | 0.025 |
| Glutathione metabolism | 102 | 536 | 0.028 |
| Benzoxazinoid biosynthesis | 7 | 20 | 0.029 |
| Glycine; serine and threonine metabolism | 94 | 495 | 0.035 |
| Diterpenoid biosynthesis | 11 | 39 | 0.036 |
| Alanine; aspartate and glutamate metabolism | 11 | 41 | 0.05 |
| **Late acclimation -> De-acclimation** | | | |
| **Plant-pathogen interaction** | 200 | 603 | 5.30E-19 |
| **Pentose and glucuronate interconversions** | 213 | 764 | 1.40E-11 |
| **Plant hormone signal transduction** | 181 | 682 | 1.80E-08 |
| **Photosynthesis** | 45 | 136 | 1.30E-05 |
| **Photosynthesis - antenna proteins** | 19 | 40 | 1.50E-05 |
| Aminobenzoate degradation | 30 | 82 | 4.00E-05 |
| Fructose and mannose metabolism | 71 | 258 | 7.60E-05 |
| Flavonoid biosynthesis | 29 | 81 | 8.50E-05 |
| Glucosinolate biosynthesis | 43 | 139 | 0.00011 |
| Alanine; aspartate and glutamate metabolism | 16 | 41 | 0.0011 |
| Glyoxylate and dicarboxylate metabolism | 20 | 57 | 0.0013 |
| alpha-Linolenic acid metabolism | 15 | 39 | 0.0018 |
| Phenylpropanoid biosynthesis | 79 | 326 | 0.0021 |
| Glycerolipid metabolism | 58 | 228 | 0.0024 |
| Phenylalanine metabolism | 9 | 19 | 0.0029 |
| Zeatin biosynthesis | 16 | 45 | 0.0033 |
| Starch and sucrose metabolism | 62 | 251 | 0.0036 |
| Brassinosteroid biosynthesis | 10 | 23 | 0.0037 |
| Mineral absorption | 38 | 142 | 0.005 |
| Pentose phosphate pathway | 28 | 98 | 0.0056 |
| Glycine; serine and threonine metabolism | 110 | 495 | 0.007 |
| Glutathione metabolism | 117 | 536 | 0.0098 |
| Biosynthesis of unsaturated fatty acids | 13 | 39 | 0.014 |
| Benzoxazinoid biosynthesis | 8 | 20 | 0.016 |
| Arachidonic acid metabolism | 13 | 40 | 0.017 |
| Carbon fixation in photosynthetic organisms | 5 | 11 | 0.031 |
| Diterpenoid biosynthesis | 12 | 39 | 0.033 |
| Cyanoamino acid metabolism | 45 | 195 | 0.035 |
| Methane metabolism | 14 | 48 | 0.035 |
| Nitrogen metabolism | 22 | 86 | 0.043 |
| Protein digestion and absorption | 18 | 68 | 0.047 |
| Carotenoid biosynthesis | 16 | 59 | 0.048 |
| **De-acclimation -> Non-acclimation** | | | |
| **Plant-pathogen interaction** | 78 | 603 | 1.10E-07 |
| **Phenylpropanoid biosynthesis** | 47 | 326 | 1.50E-06 |
| **Diterpenoid biosynthesis** | 11 | 39 | 4.10E-05 |
| **Cell adhesion molecules** | 11 | 46 | 0.00021 |
| **Glucosinolate biosynthesis** | 21 | 139 | 0.00052 |
| Pentose and glucuronate interconversions | 77 | 764 | 0.00062 |
| Plant hormone signal transduction | 70 | 682 | 0.00064 |
| Flavonoid biosynthesis | 14 | 81 | 0.0011 |
| Aminobenzoate degradation | 14 | 82 | 0.0013 |
| Zeatin biosynthesis | 8 | 45 | 0.01 |
| Chlorocyclohexane and chlorobenzene degradation | 7 | 37 | 0.011 |
| Novobiocin biosynthesis | 5 | 21 | 0.012 |
| Protein digestion and absorption | 10 | 68 | 0.016 |
| Glycine; serine and threonine metabolism | 45 | 495 | 0.033 |
| Glycerolipid metabolism | 23 | 228 | 0.04 |
| alpha-Linolenic acid metabolism | 6 | 39 | 0.047 |
| ABC transporters | 15 | 138 | 0.049 |
| Fructose and mannose metabolism | 25 | 258 | 0.049 |
| MAPK signaling pathway - yeast | 5 | 30 | 0.05 |

**Supplementary** **Table S3.** The expression of genes encoding ‘LEA proteins (including dehydrins)’ and ‘molecular chaperones/heat shock proteins (HSPs)’ during transition to different acclimation phases.

| **Contig_ID** | **Annotation** | **NA-> EA** | **EA-> LA** | **NA-> LA** | **LA-> DA** | **DA-> NA** |
| --- | --- | --- | --- | --- | --- | --- |
| **Genes encoding late embryogenesis abundant proteins** | | | | | | |
| CL888Contig2 | >XP_028057985.1 Late embryogenesis abundant protein Lea14-A [*Camellia sinensis*] | 5.23 | 2.84 | 8.02 | -8.94 |  |
| CL2066Contig1 | >AST13898.1 Late embryogenisis abundant protein 13 [*Betula platyphylla*] | 5.10 |  | 6.68 | -9.01 |  |
| CL10Contig8 | >XP_028191916.1 Late embryogenesis abundant protein 2-like [*Glycine soja*] | 4.89 | 2.18 | 6.97 | -6.99 |  |
| C83592 | >XP_022036052.1 Late embryogenesis abundant protein; group 3-like [*Helianthus annuus*] | 4.44 |  | 4.62 | -8.94 | 4.35 |
| CL6246Contig1 | >XP_022991963.1 Late embryogenesis abundant protein D-34-like [*Cucurbita maxima*] | 4.07 |  | 5.65 | -7.12 |  |
| CL714Contig2 | >XP_007222687.1 Late embryogenesis abundant protein D-29 [*Prunus persica*] | 3.21 |  | 4.25 | -8.78 | 4.55 |
| CL714Contig1 | >XP_022870395.1 Late embryogenesis abundant protein D-29-like [*Olea europaea* var. *sylvestris*] | 2.90 |  | 3.75 | -8.53 | 4.81 |
| CL10Contig9 | >AEY78063.1 Late embryogenesis abundant protein 1 [*Deschampsia* *antarctica*] | 2.50 |  | 3.25 | -5.17 |  |
| CL10Contig6 | >XP_018490726.1 Late embryogenesis abundant protein 76-like isoform X2 [*Raphanus sativus*] | 2.44 |  | 3.29 | -4.96 |  |
| C158266 | >P83442.1 RecName: Full= Late embryogenesis abundant protein Dc3 | 2.12 |  | 2.78 | -6.48 | 3.70 |
| C195978 | >PSS07444.1 Late embryogenesis abundant protein; LEA-14 protein; partial [*Actinidia chinensis* var. *chinensis*] | -2.21 |  | -3.21 | 3.28 |  |
| C157102 | >PSS34284.1 Late embryogenesis abundant protein [*Actinidia chinensis* var. *chinensis*] | -2.26 | 2.70 |  |  |  |
| CL2943Contig1 | >PSS33019.1 Late embryogenesis abundant protein [*Actinidia chinensis* var. *chinensis*] | -2.60 |  |  |  |  |
| C131648 | >XP_027168518.1 Late embryogenesis abundant protein Dc3-like [*Coffea eugenioides*] |  | 8.63 | 8.65 | -8.67 |  |
| scaffold14920 | >PSS04406.1 Late embryogenesis abundant protein [*Actinidia chinensis* var. *chinensis*] |  | 6.30 | 6.33 | 2.17 | -8.61 |
| CL10490Contig1 | >XP_017251437.1 Late embryogenesis abundant protein Dc3 [*Daucus carota* subsp. *sativus*] |  | 6.00 | 6.02 | -6.04 |  |
| CL10Contig5 | >ADC55280.1 LEA3 protein [*Ampelocalamus calcareus*] |  | 2.01 | 2.88 | -3.80 |  |
| C126702 | >PSS03992.1 Late embryogenesis abundant protein [*Actinidia chinensis* var. *chinensis*] |  |  |  | 6.83 | -6.90 |
| scaffold12108 | >PSS11360.1 Late embryogenesis abundant protein [*Actinidia chinensis* var. *chinensis*] |  |  |  | 2.61 |  |
| scaffold15036 | >XP_028070429.1 Late embryogenesis abundant protein At1g64065-like [*Camellia sinensis*] |  |  | -3.78 | 3.24 |  |
| scaffold9407 | >XP_030513935.1 Late embryogenesis abundant protein Lea5-D-like [*Rhodamnia argentea*] |  |  |  | 2.06 |  |
| **Genes encoding dehydrins** | | | | | | |
| C136590 | >AAB84258.1 Dehydrin 1 [*Vaccinium corymbosum*] | 4.05 |  | 5.34 | -8.11 | 2.80 |
| CL257Contig3 | >AGI36547.1 Dehydrin 2 [*Rhododendron catawbiense*] | 4.00 |  | 5.16 | -2.79 | -2.37 |
| CL4Contig7 | >PRQ47518.1 Putative dehydrin [*Rosa chinensis*] | 3.77 |  | 4.91 | -8.22 | 3.35 |
| **Genes encoding molecular chaperones** | | | | | | |
| C198490 | >PHT34937.1 Chaperone protein dnaJ 3 [*Capsicum baccatum*] | 7.72 | -7.61 |  |  |  |
| C144398 | >AQK64136.1 Chaperone protein dnaJ 3 [*Zea mays*] |  | 7.16 | 3.09 |  |  |
| CL7451Contig1 | >PSS11985.1 Chaperone protein like [*Actinidia chinensis* var. *chinensis*] |  |  | 4.79 |  |  |
| scaffold12410 | >PSR93406.1 BAG family molecular chaperone regulator like [*Actinidia chinensis* var. *chinensis*] | -2.166 |  | -3.23 |  |  |
| C46650 | >XP_021821518.1 Chaperone protein ClpB1-like [*Prunus avium*] | -4.45 |  |  |  |  |
| scaffold17853 | >PIN17372.1 Copper chaperone [*Handroanthus impetiginosus*] | -4.92 |  | -5.10 | 3.02 | 2.11 |
| scaffold15092 | >XP_028104345.1 BAG family molecular chaperone regulator 7 [*Camellia sinensis*] | -7.60 |  | -3.89 | 5.50 |  |
| C201902 | >XP_028115408.1 Chaperone protein ClpD; chloroplastic-like isoform X2 [*Camellia sinensis*] |  | 2.41 | 3.78 |  | -2.20 |
| C202030 | >XP_004235966.1 Chaperone protein ClpB1 [*Solanum lycopersicum*] |  |  | 11.95 | -11.97 |  |
| scaffold9822 | >XP_028122098.1 Chaperone protein dnaJ C76; chloroplastic isoform X1 [*Camellia sinensis*] |  |  | 2.38 | -2.03 |  |
| C129628 | >PSS13848.1 Chaperone protein like [*Actinidia chinensis* var. *chinensis*] |  |  |  | 2.19 |  |
| C34210 | >XP_027356597.1 Chaperone protein dnaJ 11; chloroplastic-like [*Abrus precatorius*] |  |  |  | 2.01 |  |
| CL2068Contig1 | >PSR85431.1 Chaperone protein dnaJ 11 like [*Actinidia chinensis* var. *chinensis*] |  |  |  | 2.19 |  |
| scaffold1209 | >XP_028116989.1 Chaperone protein dnaJ 11; chloroplastic-like [*Camellia sinensis*] |  |  |  | 2.15 |  |
| C138614 | >KRM80698.1 ATP-dependent chaperone ClpB [*Lactobacillus coleohominis* DSM 14060] |  |  |  | 8.88 | -8.96 |
| C173312 | >PSS29509.1 Chaperone protein dnaJ 11 like [*Actinidia chinensis* var. *chinensis*] |  |  |  | 3.14 | -3.54 |
| CL5510Contig1 | >XP_020965904.1 Chaperone protein dnaJ 8; chloroplastic-like; partial [*Arachis ipaensis*] |  |  |  | 2.22 | -2.23 |
| **Genes encoding HSPs** | | | | | | |
| scaffold7636 | >XP_016548950.1 Predicted: Stromal 70 kDa heat shock-related protein; chloroplastic-like [*Capsicum annuum*] | 14.61 |  | 13.11 | -13.13 |  |
| C201602 | >PIN20286.1 Molecular chaperone (HSP90 family) [*Handroanthus impetiginosus*] | 13.72 | -13.62 |  |  |  |
| C178956 | >CAA12387.1 HSP 20.1 protein [*Solanum peruvianum*] | 12.97 | -12.88 |  |  |  |
| C201434 | >PHT45127.1 HSP 90-1 [*Capsicum baccatum*] | 12.92 |  | 11.57 | -11.59 |  |
| C201222 | >PHU16979.1 Heat shock cognate 70 kDa protein [*Capsicum chinense*] | 8.81 | -8.69 |  |  |  |
| C200382 | >PHT46761.1 Heat shock cognate 70 kDa protein [*Capsicum baccatum*] | 8.75 | -8.64 |  |  |  |
| scaffold11299 | >PSS17576.1 HSP 20-like chaperone protein [*Actinidia chinensis* var. *chinensis*] | 8.20 | 4.98 | 13.09 | -9.27 |  |
| scaffold18493 | >XP_012478818.1 Predicted: HSP 83-like isoform X1 [*Gossypium raimondii*] | 8.14 | -8.03 |  |  |  |
| scaffold18492 | >XP_006348634.1 Predicted: HSP 90-5; chloroplastic [*Solanum tuberosum*] | 7.97 |  | 12.01 | -12.03 |  |
| CL1390Contig2 | >XP_016544958.1 Predicted: Heat shock cognate protein 80-like isoform X1 [*Capsicum annuum*] | 7.84 | -7.73 |  |  |  |
| scaffold17389 | >PHT41715.1 Heat shock 70 kDa protein; mitochondrial [*Capsicum baccatum*] | 5.52 | -5.41 |  |  |  |
| C182622 | >AIX87538.1 HSP 21 [*Lycium ruthenicum*] | 5.41 | -5.31 |  |  |  |
| C105036 | >ABW69466.1 HSP 70; partial [*Nannizzia racemosa*] | 5.20 | -5.11 |  | 4.85 | -4.92 |
| C154724 | >PSS15503.1 Heat shock factor protein [*Actinidia chinensis* var. *chinensis*] | 3.23 |  | 4.26 | -7.89 |  |
| CL91Contig2 | >PSR96855.1 Heat shock 70 protein [*Actinidia chinensis* var. *chinensis*] | -4.98 | 5.11 |  |  |  |
| C159858 | >PSS32961.1 Small heat shock protein [*Actinidia chinensis* var. *chinensis*] |  | 10.04 | 6.88 | -3.32 | -3.39 |
| CL4145Contig1 | >PSS10321.1 Heat shock 70 protein; partial [*Actinidia chinensis* var. *chinensis*] |  | 5.86 |  |  |  |
| scaffold8734 | >AFN89710.1 HSP 21.4 [*Primula forrestii*] |  | 5.13 | 3.17 |  |  |
| scaffold8782 | >XP_028060152.1 heat shock factor protein HSF30-like [*Camellia sinensis*] |  | 4.71 | 7.34 | -10.84 |  |
| C124642 | >BAF62730.1 HSP 70; partial [*Paracoccidioides brasiliensis*] |  | 4.52 |  |  | -3.42 |
| C154878 | >XP_028243065.1 17.3 kDa class I heat shock protein [*Glycine soja*] |  | 3.78 | 3.90 |  | -2.75 |
| scaffold15862 | >XP_030491945.1 Heat shock 70 kDa protein-like [*Cannabis sativa*] |  | 3.34 | 2.50 |  |  |
| C117264 | >WP_089454972.1 HSP 20 family protein; partial [*Escherichia coli*] |  | 3.31 |  |  |  |
| CL2209Contig1 | >XP_009334702.1 Predicted: 17.1 kDa class II heat shock protein-like [*Pyrus* x *bretschneideri*] |  | 3.23 |  |  |  |
| CL1664Contig1 | >XP_022143736.1 Heat shock cognate 70 kDa protein 2 [*Momordica charantia*] |  | -2.02 | -2.01 |  |  |
| C180204 | >XP_028124611.1 21.7 kDa class VI heat shock protein-like [*Camellia sinensis*] |  | -2.17 | -2.91 | 2.63 |  |
| CL9261Contig1 | >XP_023925105.1 Heat shock 70 kDa protein 6; chloroplastic-like [*Quercus suber*] |  |  | 3.95 | -3.97 |  |
| scaffold10418 | >XP_012071995.1 Small heat shock protein; chloroplastic [*Jatropha curcas*] |  |  | 3.70 | -2.60 |  |
| scaffold16238 | >XP_002526446.1 Stromal 70 kDa heat shock-related protein; chloroplastic [*Ricinus communis*] |  |  | 2.73 | -2.64 |  |
| CL4902Contig1 | >PHT85973.1 Heat shock 70 kDa protein 8 [*Capsicum annuum*] |  |  | 2.57 | -2.23 |  |
| C165312 | >RVW41347.1 18.2 kDa class I heat shock protein [*Vitis vinifera*] |  |  | -2.15 | 3.15 |  |
| CL4052Contig1 | >XP_007129028.3 Low quality protein: Heat shock cognate 71 kDa protein [*Physeter catodon*] |  |  | -2.48 |  |  |
| C131272 | >AAC84134.1 HSP; partial [*Cichorium intybus*] |  |  | -2.63 | -4.24 | 6.98 |
| C109546 | >XP_021724478.1 HSP 90-6; mitochondrial-like isoform X2 [*Chenopodium quinoa*] |  |  | -2.82 | 2.83 |  |
| C95838 | >THW06196.1 HSP 70 [*Aureobasidium pullulans*] |  |  | -2.94 |  |  |
| scaffold16652 | >APD25541.1 Heat shock factor B3 [*Rhododendron calophytum*] |  |  |  | 2.66 | -2.15 |
| CL4502Contig1 | >OMO56700.1 Heat shock factor (HSF)-type; DNA-binding protein [*Corchorus capsularis*] |  |  |  |  | -2.67 |
| C122092 | >ORY22434.1 HSP 9/12 [*Naematelia encephala*] |  |  |  | 2.39 |  |
| scaffold6925 | >PSS11530.1 Small HSP [*Actinidia chinensis* var. *chinensis*] |  |  |  | -3.02 |  |
| CL892Contig1 | >QBS00797.1 HSP 70; partial [*Agave sisalana*] |  |  |  | -3.67 | 3.36 |
| C113230 | >TKA49864.1 Heat shock protein SSB; partial [*Friedmanniomyces simplex*] |  |  |  | 4.77 | -4.84 |
| C150388 | >XP_008085807.1 HSP 12 [*Glarea lozoyensis*] |  |  |  | 3.25 | -7.75 |
| C201162 | >XP_019232766.1 Predicted: HSP 82 [*Nicotiana attenuata*] |  |  |  | -11.67 | 12.57 |

**Supplementary** **Table S4.** The expression of genes associated with ‘photosynthesis’ during the transition to different acclimation phases.

| **Contig_ID** | **Annotation** | **NA-> EA** | **EA-> LA** | **NA-> LA** | **LA-> DA** | **DA->**  **NA** |
| --- | --- | --- | --- | --- | --- | --- |
| **Photosynthesis** | | | | | | |
| C163448 | >XP_004248376.1 Photosynthetic NDH subunit of lumenal location 3; chloroplastic [*Solanum lycopersicum*] | -4.24 |  | -6.20 | 6.77 |  |
| CL8005Contig1 | >XP_021850776.1 Photosystem I reaction center subunit N; chloroplastic [*Spinacia oleracea*] |  | -3.68 | -4.90 | 6.46 |  |
| C153656 | >PHT30123.1 Photosystem I subunit O [*Capsicum baccatum*] | -2.39 |  | -4.42 | 5.13 |  |
| CL8314Contig1 | >XP_007032986.2 Predicted: PsbQ-like protein 3; chloroplastic [*Theobroma cacao*] |  | -3.00 | -4.52 | 4.66 |  |
| scaffold18331 | >GAV76409.1 PSI_PSAK domain-containing protein [*Cephalotus follicularis*] | -2.21 |  | -3.03 | 4.60 |  |
| C184160 | >XP_023886996.1 Photosystem I reaction center subunit N; chloroplastic [*Quercus suber*] |  |  | -3.58 | 4.50 |  |
| C27208 | >PSR95757.1 PsbP-like protein [*Actinidia chinensis* var. *chinensis*] |  | -4.36 | -5.04 | 4.41 |  |
| C24690 | >ADD31525.1 ATP synthase CF0 subunit I protein (chloroplast) [*Rhododendron simsii*] |  |  |  | 4.32 | -4.39 |
| C70927 | >OTG29929.1 Putative cytochrome b6/f complex; subunit IV [*Helianthus annuus*] |  |  |  | 4.20 |  |
| scaffold14317 | >XP_009370977.1 Predicted: Photosystem I reaction center subunit IV B; chloroplastic [*Pyrus* x *bretschneideri*] | -2.06 |  | -3.58 | 4.14 |  |
| CL4491Contig1 | >XP_002510603.1 Plastocyanin B'/B'' [*Ricinus communis*] |  |  | -3.44 | 4.04 |  |
| scaffold15489 | >XP_028126933.1 Photosynthetic NDH subunit of lumenal location 1; chloroplastic [*Camellia sinensis*] |  | -2.08 | -3.57 | 3.84 |  |
| CL1842Contig1 | >PSS01446.1 Photosystem I reaction center subunit II like [*Actinidia chinensis* var. *chinensis*] |  |  | -3.19 | 3.74 |  |
| C164326 | >VVA30744.1 Predicted: photosynthetic NDH subunit of lumenal [*Prunus dulcis*] |  |  | -3.22 | 3.68 |  |
| CL867Contig1 | >XP_021907147.1 Photosystem I reaction center subunit XI; chloroplastic [*Carica papaya*] |  |  | -2.77 | 3.23 |  |
| CL3205Contig1 | >AZQ19329.1 Photosystem II oxygen-evolving complex protein 2 precursor [*Populus tomentosa*] |  |  | -2.45 | 3.19 |  |
| C80093 | >XP_030551009.1 Cytochrome b6-f complex iron-sulfur subunit; chloroplastic-like [*Rhodamnia argentea*] |  |  |  | 3.17 |  |
| scaffold9299 | >XP_028054894.1 Photosystem I reaction center subunit VI; chloroplastic-like [*Camellia sinensis*] |  |  | -2.63 | 3.15 |  |
| CL4994Contig1 | >PSS30786.1 Oxygen-evolving enhancer protein like [*Actinidia chinensis* var. *chinensis*] |  |  | -2.56 | 3.13 |  |
| CL10793Contig1 | >XP_022877746.1 Photosynthetic NDH subunit of lumenal location 2; chloroplastic [*Olea europaea* var. *sylvestris*] |  |  | -2.89 | 3.10 |  |
| CL4568Contig1 | >XP_028127603.1 ATP synthase delta chain; chloroplastic [*Camellia sinensis*] |  |  | -2.33 | 2.81 |  |
| scaffold5826 | >XP_027148083.1 Photosystem I reaction center subunit IV B; chloroplastic-like [*Coffea eugenioides*] |  |  | -2.06 | 2.76 |  |
| CL584Contig1 | >XP_028123539.1 Ferredoxin [*Camellia sinensis*] |  |  | -2.49 | 2.74 |  |
| CL2310Contig1 | >PSR96332.1 Cytochrome b6-f complex iron-sulfur subunit 1 like [*Actinidia chinensis* var. *chinensis*] |  |  | -2.36 | 2.65 |  |
| CL838Contig2 | >XP_022845187.1 ATP synthase subunit b'; chloroplastic [*Olea europaea* var. *sylvestris*] |  |  | -2.31 | 2.55 |  |
| C165124 | >XP_028108831.1 PsbP domain-containing protein 7; chloroplastic isoform X2 [*Camellia sinensis*] |  |  |  | 2.34 |  |
| scaffold12569 | >XP_022025772.1 Photosystem I reaction center subunit III; chloroplastic [*Helianthus annuus*] |  |  |  | 2.34 |  |
| scaffold4423 | >XP_028113066.1 NifU-like protein 2; chloroplastic [*Camellia sinensis*] |  |  |  | 2.29 |  |
| C114230 | >PWA99867.1 Photosystem I reaction center subunit V [*Artemisia annua*] |  |  |  | 2.26 |  |
| scaffold13335 | >XP_028059182.1 PsbP-like protein 1; chloroplastic isoform X1 [*Camellia sinensis*] |  |  |  | 2.19 |  |
| CL682Contig1 | >BAH20433.1 AT4G04640; partial [*Arabidopsis thaliana*] |  |  |  | 2.05 |  |
| scaffold8965 | >YP_009517995.1 Photosystem I P700 apoprotein A1 (chloroplast) [*Physalis angulata*] | 4.75 |  | 3.49 | -2.89 |  |
| C187726 | >PHT32220.1 ATP synthase delta chain; chloroplastic [*Capsicum baccatum*] |  |  | 10.94 | -10.96 |  |
| C42532 | >AFU95988.1 PetB; partial (chloroplast) [*Humiria balsamifera*] |  |  |  | -3.95 |  |
| scaffold16822 | >OTG27374.1 Putative photosynthetic reaction centre; L/M; Photosystem antenna protein-like protein [*Helianthus annuus*] |  |  |  | -5.23 | 5.58 |
| C192948 | >XP_016491746.1 Predicted: oxygen-evolving enhancer protein 1; chloroplastic-like [*Nicotiana tabacum*] |  |  |  | -12.63 | 13.47 |
| C64188 | >YP_009676316.1 ATP synthase CF0 subunit I (chloroplast) [*Sclerocarya birrea*] | -4.74 |  |  |  |  |
| C109972 | >XP_027089834.1 ATP synthase delta chain; chloroplastic-like [*Coffea arabica*] |  |  | -2.03 |  |  |
| C163366 | >XP_016552487.1 Predicted: Photosystem I reaction center subunit N; chloroplastic [*Capsicum annuum*] | 8.06 | -7.95 |  |  |  |
| C170220 | >NP_001289499.1 Photosystem I reaction center subunit VI-1; chloroplastic-like precursor [*Nicotiana sylvestris*] | 7.51 | -7.40 |  |  |  |
| C179872 | >XP_006356675.1 Predicted: Photosystem I reaction center subunit II; chloroplastic [*Solanum tuberosum*] | 14.13 | -14.03 |  |  |  |
| C185778 | >XP_016559312.1 Predicted: Photosystem I reaction center subunit III; chloroplastic-like [*Capsicum annuum*] | 8.73 | -8.61 |  |  |  |
| C195128 | >XP_016558935.1 Predicted: Oxygen-evolving enhancer protein 1; chloroplastic [*Capsicum annuum*] | 13.76 | -13.67 |  |  |  |
| scaffold6299 | >PHT42714.1 Oxygen-evolving enhancer protein 2; chloroplastic [*Capsicum baccatum*] | 8.01 | -7.89 |  |  |  |
| C193744 | >YP_009578905.1 ATP synthase CF0 A subunit (chloroplast) [*Taxus florinii*] |  |  | 2.88 |  |  |
| C114706 | >KEH42763.1 ATP synthase protein [*Medicago truncatula*] |  |  | 5.38 |  |  |
| scaffold7023 | >PSR98576.1 CBS domain-containing protein [*Actinidia chinensis* var. *chinensis*] |  | 2.49 |  |  |  |
| **Carbon fixation in photosynthetic organisms** | | | | | | |
| C132406 | >PSS32550.1 Sedoheptulose-1;7-bisphosphatase [*Actinidia chinensis* var. *chinensis*] |  |  | -2.57 | 2.76 |  |
| C177214 | >RHN45839.1 Putative glyceraldehyde-3-phosphate dehydrogenase (NADP(+)) (phosphorylating) [*Medicago truncatula*] |  |  |  | 2.35 |  |
| CL103Contig1 | >APB08587.1 GAPDH [*Rhododendron molle*] |  | -2.05 | -3.48 | 4.27 |  |
| CL227Contig1 | >ABR25696.1 Chloroplast phosphoribulokinase precursor; partial [*Oryza sativa* *Indica*] |  |  |  | 6.30 | -6.37 |
| scaffold14149 | >XP_015076224.1 Sedoheptulose-1;7-bisphosphatase; chloroplastic [*Solanum pennellii*] | 8.08 | -7.97 |  |  |  |
| **Photosynthesis - antenna proteins** | | | | | | |
| C168364 | >XP_010474550.1 Predicted: Chlorophyll a-b binding protein 3; chloroplastic-like [*Camelina sativa*] | -3.44 |  | -5.67 | 6.93 |  |
| C193248 | >XP_002284493.1 Predicted: Chlorophyll a-b binding protein 13; chloroplastic [*Vitis vinifera*] | -2.25 |  | -4.05 | 4.82 |  |
| CL1261Contig1 | >PSR93011.1 Chlorophyll a-b binding protein like [*Actinidia chinensis* var. *chinensis*] | -2.54 |  | -4.26 | 5.32 |  |
| CL1261Contig2 | >XP_019224862.1 Predicted: Chlorophyll a-b binding protein 6A; chloroplastic [*Nicotiana attenuata*] | -2.46 |  | -3.61 | 4.37 |  |
| CL2699Contig1 | >RVW99274.1 Chlorophyll a-b binding protein; chloroplastic [*Vitis vinifera*] |  |  | -2.60 | 3.30 |  |
| CL2851Contig1 | >XP_020689536.1 Chlorophyll a-b binding protein 5; chloroplastic [*Dendrobium catenatum*] |  |  | -2.58 | 3.57 |  |
| CL4061Contig1 | >XP_024984456.1 Photosystem I chlorophyll a/b-binding protein 3-1; chloroplastic-like [*Cynara cardunculus* var. *scolymus*] |  |  | -2.08 | 2.75 |  |
| CL466Contig1 | >XP_021592964.1 Chlorophyll a-b binding protein P4; chloroplastic [*Manihot esculenta*] | -2.60 |  | -4.46 | 5.18 |  |
| CL7647Contig1 | >PSS02042.1 Photosystem I chlorophyll a/b-binding protein [*Actinidia chinensis* var. *chinensis*] |  | -2.25 | -3.09 | 3.25 |  |
| CL7699Contig1 | >PSR99766.1 Photosystem I chlorophyll a/b-binding protein like [*Actinidia chinensis* var. *chinensis*] | -2.44 |  | -4.98 | 5.57 |  |
| CL8239Contig1 | >XP_006360010.1 Predicted: Chlorophyll a-b binding protein 8; chloroplastic [*Solanum tuberosum*] |  |  | -4.68 |  | 4.74 |
| scaffold13192 | >PHT29505.1 Chlorophyll a-b binding protein CP26; chloroplastic [*Capsicum baccatum*] | 9.19 | -9.08 |  |  |  |
| C181576 | >NP_001317571.1 Chlorophyll a-b binding protein Cab9 [*Solanum lycopersicum*] |  |  | 9.75 | -12.04 |  |
| C187898 | >PHU18994.1 Chlorophyll a-b binding protein 3C; chloroplastic [*Capsicum chinense*] | 8.84 | -8.72 |  |  |  |
| C190634 | >XP_016580520.1 Predicted: Chlorophyll a-b binding protein 5; chloroplastic-like [*Capsicum annuum*] | 11.51 | -9.66 |  | -4.39 |  |
| C191120 | >XP_009588563.1 Predicted: Chlorophyll a-b binding protein CP29.2; chloroplastic [*Nicotiana tomentosiformis*] | 8.92 | -8.80 |  |  |  |
| C191674 | >NP_001316908.1 Chlorophyll a-b binding protein CAB11 [*Solanum lycopersicum*] | 13.78 | -13.69 |  |  |  |
| **Porphyrin and chlorophyll metabolism** | | | | | | |
| C114724 | >PSR93156.1 Tripartite terminase [*Actinidia chinensis* var. *chinensis*] |  |  | -3.65 | 4.80 |  |
| C136208 | >XP_028106569.1 Light-harvesting complex-like protein OHP1; chloroplastic [*Camellia sinensis*] | -3.94 |  | -4.03 | 4.51 |  |
| C152094 | >PON88127.1 Chlorophyll a/b binding protein domain containing protein [*Trema orientale*] |  |  |  | 2.14 |  |
| C182644 | >PSR93273.1 Methylesterase [*Actinidia chinensis* var. *chinensis*] |  |  |  | 2.25 |  |
| CL2710Contig1 | >XP_022997102.1 Glutamyl-tRNA reductase 1; chloroplastic [*Cucurbita maxima*] | -3.55 |  | -4.33 | 5.78 |  |
| CL476Contig2 | >PSS29927.1 Magnesium-protoporphyrin IX monomethyl ester [oxidative] cyclase [*Actinidia chinensis* var. *chinensis*] |  |  |  | 2.39 |  |
| CL5063Contig1 | >PSR98619.1 Magnesium-chelatase subunit ChlI like [*Actinidia chinensis* var. *chinensis*] |  |  | -2.07 | 2.01 |  |
| CL7045Contig1 | >XP_020219459.1 Magnesium-protoporphyrin IX monomethyl ester [oxidative] cyclase; chloroplastic [*Cajanus cajan*] |  |  |  | 2.36 |  |
| CL9195Contig1 | >XP_002515173.1 Protoporphyrinogen oxidase 1; chloroplastic [*Ricinus communis*] |  | -2.79 | -2.73 | 3.53 |  |
| CL9314Contig1 | >PSS21167.1 Magnesium-chelatase subunit ChlH like [*Actinidia chinensis* var. *chinensis*] |  |  |  | 2.49 |  |
| scaffold18991 | >PSS36344.1 Magnesium-chelatase subunit ChlH like [*Actinidia chinensis* var. *chinensis*] |  |  |  | 2.39 |  |
| C141868 | >PSS36497.1 Zinc finger protein [*Actinidia chinensis* var. *chinensis*] | -2.43 |  |  |  |  |
| C52830 | >PSR86546.1 Pheophorbide a oxygenase [*Actinidia chinensis* var. *chinensis*] | 2.05 |  | 2.14 |  |  |
| CL102Contig2 | >RVW78060.1 Laccase-14 [*Vitis vinifera*] |  |  |  | -3.03 | 2.04 |
| C129016 | >KEH44395.1 2Fe-2S iron-sulfur cluster-binding domain protein [*Medicago truncatula*] |  | 4.53 |  |  |  |
| C139330 | >OAY72146.1 Soluble inorganic pyrophosphatase 4 [*Ananas comosus*] |  |  |  |  | -2.71 |
